# Both local stability and dispersal contribute to metacommunity sensitivity to asynchronous habitat availability

**DOI:** 10.1038/s41598-024-56632-y

**Published:** 2024-03-15

**Authors:** Pablo Moisset de Espanés, Rodrigo Ramos-Jiliberto

**Affiliations:** 1 Centro de Biotecnología y Bioingeniería, Universidad de Chile, Av. Beaucheff 851, Santiago, Chile; 2 GEMA Center for Genomics, Ecology and Environment, Universidad Mayor, Camino La Pirámide 5750, Huechuraba, Santiago Chile

**Keywords:** Local stability, Metacommunity dynamics, Dynamic landscape, Intermittent habitats, Ecological networks, Modeling, Biodiversity, Community ecology, Ecological modelling, Ecological networks, Population dynamics, Theoretical ecology

## Abstract

The stability of isolated communities depends on the complexity of their foodwebs. However, it remains unclear how local stability interacts with dispersal in multitrophic metacommunities to shape biodiversity patterns. This lack of understanding is deeper in the more realistic frame of landscapes that exhibit non-trivial and time-varying structures. Therefore, in this study, we aim to evaluate the influence of local stabilizing factors versus dispersal in determining the sensitivity of metacommunity biodiversity to increasing asynchrony of site availability. Additionally, we assess the role of foodweb complexity and landscape structure as modulating factors. To accomplish our goals we developed a model based on random matrices for local communities, which are linked by stochastic dispersal over explicit dynamic landscapes. We ran numerical simulations and computed the effect sizes of foodweb temperature, self-limitation, dispersal ability, and all pairwise combinations, on the sensitivity of biodiversity to landscape asynchrony. In our experiments we explored gradients of species richness, foodweb connectance, number of sites, and landscape modularity. Our results showed that asynchrony among site availability periods reduced $\alpha$-diversity and increased $\beta$-diversity. Asynchrony increased $\gamma$-diversity at high dispersal rates. Both local and regional stabilizing factors determined the sensitivity of metacommunities to landscape asynchrony. Local factors were more influential in landscapes with fewer sites and lower modularity, as well as in metacommunities composed of complex foodwebs. This research offers insights into the dynamics of metacommunities in dynamic landscapes, providing valuable knowledge about the interplay between local and regional factors in shaping ecological stability and species persistence.

## Introduction

During past decades, there have been remarkable advances in the understanding of the interrelationship between ecological stability and species diversity^1–4^. These advances have been mostly reached considering local, closed ecological communities. However, many natural communities are open to regional influences driven by the dispersal of individuals. The advent of the metacommunity concept^5^ enlarged the scale of analysis, incorporating the connectedness among local communities for understanding the coordinated dynamics of spatially-structured ecological networks that resemble more closely the structure of real ecosystems. Thus, the dynamics of metacommunities are understood as governed by the interplay between local processes, that take place within local communities, and regional ones, at the level of the whole landscape^5,6^. In this vein, a central topic is understanding which properties of metacommunities determine biodiversity robustness to ongoing environmental changes.

At a local level, community stability refers to the ability of a community to retain its structure after suffering a disturbance. However, there exist many metrics that capture different aspects of community stability^7,8^. The arrangement and strength of interactions among species determine the stability of communities and the likelihood of species coexistence therein. In particular, the strength of self-limitation is an important stabilizing mechanism that modulates coexistence^9,10^, along with density-dependent interspecific processes^9^. Topological network properties, such as species richness, connectance, modularity, nestedness^11^, and trophic coherence^12^, see our Methods section also shape community stability. In a spatially-structured context, the stability of local communities should be more relevant for biodiversity maintenance in loosely connected metacommunities, where regional influences are minimal^13^. However, the likelihood of species introductions from neighboring habitats may be influenced by the stability of the destination community, consequently affecting the probability of species integration into a local community after dispersal^14,15^. Therefore, local stabilizing factors may play a crucial role in shaping the collective dynamics of linked communities. However, this particular aspect has received limited attention in previous research^13,16,17^, but see.

Regional processes are governed by the movements of organisms and propagules among local communities. These dispersal movements allow the colonization and recolonization of available and reachable sites, thereby recovering low-density populations^18^. As a consequence, moderate rates of species dispersal over a landscape tends to foster local species diversity^19^, and overall metacommunity stability for species rich foodwebs^16^. The dispersal process at the metacommunity level and its consequences on biodiversity patterns may heavily depend on landscape structure, i.e., the arrangement of links among local sites through which dispersal can occur. For example, choke points with harsh conditions can hamper the dispersal of species between sub-regions of the landscape. Conversely, high connectivity among sites fosters species abundances and reduces regional extinction probability^20^. How sites are arranged into the landscape also affects species dynamics and diversity^21,22^. In addition, landscape topology also modulates the relative importance of archetypal metacommunity driving forces (e.g., species sorting, where biodiversity is governed by the matching between species’ attributes and site-specific conditions vs. mass effect, where diversity is mainly governed by the net flux of species among sites)^23^. Consequently, there has been a recent push toward incorporating explicit landscape representations in the study of metacommunity dynamics (see, for example, Borthagaray et al., 2014, 2018).

Most metacommunity models to date, indeed those that include explicit dynamics of species abundances, assume that local habitat sites maintain their properties essentially constant over time^5^. This translates into a static structure of localities (number and connectivity of sites) that compose a landscape, as well as a static set of local conditions and resources within each site. However, real spatially-structured systems deviate significantly from these idealized representations, as ecosystems often undergo pronounced changes over time in their physical structure and in local biotic and abiotic factors^24–27^. Canonical examples of dynamic landscapes (i.e. with a time-varying structure) are the systems of temporary ponds of semiarid and Mediterranean regions^28^, e.g.. These systems consist of temporary local sites, namely the ponds, that alternate between wet and dry phases, referred to hereafter as *active* and *inactive* states respectively. Dispersal of organisms can occur between active ponds connected by channels or other media. The duration of the active period varies largely among ponds because it depends on local factors such as pond capacity and drainage. On this basis, temporary ponds are classified into semi-permanent, seasonal, intermittent, or ephemeral categories^29^. In addition, the timing of activation and deactivation of local sites is largely unpredictable. Thus these pond systems can be considered to be subjected to a frequent and stochastic disturbance sensu^30^. If all sites are active at the same time, the system reduces to a metacommunity on a static landscape over the active period. However, temporal fluctuations in habitat availability are rarely perfectly synchronized among all sites within a landscape. Consequently, the active periods of two specific sites may only partially overlap or not overlap at all. This asynchrony can have a detrimental impact on biodiversity, particularly for active dispersers, as it hinders dispersal between adjacent sites. Nevertheless, moderate levels of landscape asynchrony could also yield positive effects through enhancing asynchrony in the temporal trajectories of species, thereby enhancing spatial rescue effect mechanisms^31^. We assume that our system includes one permanent site (*the mainland*) containing the regional pool of species. In a freshwater system, a permanent lake could act as a mainland.

In this study, we assess the role of local stabilizing factors, *LSFs*, (self-limitation, trophic coherence), versus regional stabilizing factors, *RSFs*, (dispersal ability) in shaping the sensitivity of metacommunity biodiversity and biomass to increasing asynchrony in sites’ active periods (hereafter *landscape asynchrony* *A*). We evaluate these effects across gradients of both regional foodweb and landscape complexity. We hypothesize that: (i) biodiversity patterns are affected by *A* (ii) both *LSFs* and *RSFs* determine metacommunity sensitivity (in diversity and biomass) to increased *A*, and (iii) both regional foodweb topology (species richness and connectance) and landscape structure (number of local sites and spatial modularity) modulate the relative contributions of *LSFs* and *RSFs* to metacommunity sensitivity to increased *A*.

To efficiently simulate systems involving these elements, we developed a model that involves (a) random spatially-explicit metacommunities embedded in dynamic landscapes containing temporary sites, (b) local community dynamics, described as Lotka-Volterra foodweb equations parameterized by linear programming, reaching equilibria instantaneously to avoid explicit population dynamics, c) stochastic dispersal among sites obeying a Markov process.

## Methods

### Landscape generation and dynamics

We represent dynamic landscapes as dynamic graphs, i.e. time-varying graphs with node-dynamics^32^. Vertices represent sites where local communities can be assembled, edges represent a non-zero probability of species dispersal between sites, and edge weights represent the Euclidean distance between two adjacent sites. Our algorithm creates connected modular landscapes by laying sites at random on a square, creating a minimum spanning tree, and then adding edges at random to reach a desired connectance. The process uses five parameters, namely $n_P$, $n_E$, $n_C$, *F*, and *x*. They represent the number of sites, the number of edges, the number of modules, the excess factor, and the distance exponent. The first three are self-explanatory. We fixed $n_E$ to $2n_P$ and $n_C$ to 5. Real parameter $F\ge 1$ regulates how tight the modules are. A value of 1 results in no discernible clustering of sites, i.e. they are distributed uniformly at random, while a high value will produce tight modules. Real parameter $x\ge 0$ controls how the distances among sites determine the probability of edges being added to the landscape. A value of zero indicates that distances between sites do not affect the probability of edges being added. A larger value favors adding shorter edges over longer ones. Thus, spatial modularity increases with both *F* and *x*. See Fig. SI1 for an example. We set $x=2$ for all our simulations. A site of a randomly chosen module is designated as *the mainland*. See “Supplementary Information”, for the detailed algorithm.

At a given time, each site *p* is in either of two states: active or inactive. Species can be present in *p* only when the site is active. The mainland is always active, and all species in the regional pool are present therein. This allows for the repopulation of sites when they become active while avoiding the complexity of representing dormant or other latent states explicitly^33^, e.g.. All the other sites transition stochastically between the two states. For a site *p*, the nominal length of the active period is randomly drawn from a uniform distribution $w_p\sim U(0.2,0.3)$. Assuming no overlapping active periods from consecutive years, the start of the active period for the year *k* is $start_{p,k} = k + z_{p,k}$, where $z_{p,k}\sim U(-A,+A)$, and parameter $A>0$ is the magnitude of asynchrony among the sites’ activation times. The end of the period will be $end_{p,k} = start_{p,k} + w_p$. To account for overlaps, we define $\textit{ACTIVE}_p = \cup _k [\textit{start}_{p,k},\textit{end}_{p,k}]$ and we say site *p* is active at time *t* if and only if $t\in \textit{ACTIVE}_p$.

### Local community dynamics

The regional pool of species and interactions is modeled as a foodweb following^34^, which extends the Preferential Prey Model^12^. Unlike the niche or the cascade models, the algorithm in^34^ creates foodwebs with varying degrees of trophic coherence, a structural property that strongly determines stability in empirical and quasi-empirical foodwebs^12^.^34^ describe trophic coherence as “a measure of how neatly food webs or other directed networks fall into well-defined trophic level.” Hence, higher trophic coherence indicates lower omnivory, with species’ trophic levels closer to integers in the food web^12^. The algorithm creates foodweb topologies from target values of species richness $n_S$, number of basal species $n_B$, number of predation links $n_L$, and foodweb temperature *T* (a surrogate for trophic coherence). We fixed the number of basal species to 20% of the species richness. We define foodweb connectance *C* as $n_L/(n_S\cdot (n_S - n_B -1)+n_B)$, i.e. the ratio between the number of present links $n_L$ and the maximum possible number of edges in a foodweb with $n_B$ basal species and no cannibals.

We assume that the dynamics of $x_{i,p}$, the biomass of species *i* at site *p*, is governed by Lotka-Volterra type equations and reaches equilibrium instantaneously. The ODEs describing local community dynamics are:

1 $$\begin{aligned} \frac{dx_{i,p}}{dt}&= \left( r_i + \sum _j M_{ij} x_{j,p}\right) x_{i,p} , \end{aligned}$$

where *i* and *j* range over all species in the pool. Elements $M_{i,j}$ of the *community matrix* *M* represent the effect of increasing population biomass of species *j* on the per unit biomass growth rate of species *i*. Parameters $r_i$’s are the intrinsic growth rates.

To define *M*, we first assign $M_{ii}=-\lambda$, where the self-regulation parameter $\lambda$ is a positive real. If there are no trophic interactions between species *i* and *j*, then $M_{ij}=0$. If *j* feeds on *i*, then $M_{ij}=-{\mathcal {X}}$, where ${\mathcal {X}}$ is drawn from a lognormal distribution, with mean 1 and a standard deviation of 0.25. Following^12^, we set $M_{ji}=0.4 {\mathcal {X}}$. To choose the values for the $r_i$’s, we solve the linear program:

2 $$\begin{aligned} \begin{aligned}{}&\max y^*\\&\quad r^*_i + \sum _j M_{ij} x^*_j=0 \end{aligned} \end{aligned}$$

subject to $x^*_i \ge y^*$, $y^* \ge 0$, $r^*_i \le \rho$ for basal species *i*, and $r^*_i \le -\mu$ for non-basal species *i*. We set $\rho =1$ to limit the intrinsic growth rate for basals. Parameter $\mu =0.01$ is the smallest possible mortality rate value for non-basal species. The decision variables are $y^*$, all the $r^*_i$’s, and all the $x^*_i$’s. Maximizing $y^*$ means maximizing the smallest species abundance at equilibrium. If the program is not feasible, then it is impossible to choose $r^*_i$’s in such a way that all the $x^*_i$’s are positive. If this happens, the foodweb is discarded and the process is repeated. If the program is feasible, we check that, at equilibrium (the values obtained for the $x^*_i$’s), the Jacobian matrix of Eq. (1) has only eigenvalues with negative real parts. If this is not the case, then the system is unstable and it is discarded to start the process again.

### Metacommunity dynamics

The state of the metacommunity is composed of discrete variables (the presence/absence of species in each site), and continuous variables (the species biomasses at each site.) Dispersal is modeled as a continuous time Markov chain. The dispersal events, i.e. individuals moving from one site to another one, are coupled with events representing the activation and deactivation of landscape sites, and with the local community dynamics. A dispersal event of a species *s* from site *p* to site *q* is only possible if sites *p* and *q* are active, *s* is present in *p* but not in *q*, and *s* is either a basal species or a consumer with at least one of its prey species present in *q*. The biomasses of all species present in *q* are recomputed as the equilibrium of Eq. (1) and set to zero for all species whose value is below an extinction threshold of 0.001. If at least one species goes extinct, equilibrium is recalculated until no further secondary extinctions occur. By assuming that local biomass dynamics occur more rapidly than dispersal among sites^35–37^, e.g., transient dynamics can be neglected. This enables us to bypass integration of Eq. (1) and to efficiently simulate large multitrophic metacommunities.

In our setting, the set of species in *p* is not altered by dispersal events originating from *p*. The *effective rate* of dispersal events between sites *p* and *q* is the ratio between the dispersal ability $a \in {\mathbb {R}}^+$ and the Euclidean distance between *p* and *q*. However, if this ratio is less than 0.1, we set the rate to zero. Although *a* could be species-specific, in this study we assume the same value of *a* for all species. Site deactivation drives all species’ biomasses in that site to zero. The change of the discrete state variables and the dispersal/activation/deactivation/extinction events are simulated by using use a variant of the first-reaction method^38^, in the framework of dynamic Monte Carlo methods. After each migration event, we compute the continuous equilibrium biomasses using linear algebra on Eq. (1) For the details on the metacommunity simulation algorithm, see “Supplementary Information”.

### Experimental design

As main predictor variables, we chose *T* and $\lambda$ as LSFs, and *a* as the RSF. Variables *T* and $\lambda$ determine local stability, while *a* is the canonical metacommunity attribute at a regional level. Parameters *T* and $\lambda$ were set to 7 evenly spaced values between 0 and 1.2, and 6 evenly spaced values between -1.0 and -1/3, respectively. Parameter *a* was set to 5 logarithmically-spaced values between 30 and 3000. The predictors’ values were chosen based on preliminary tests, that shed light on the range of predictor values that generate noticeable variation in response variables.Regarding landscape parameters, we used $n_P= 25, 50, 100$ and $F=5, 10, 50, 75$. For foodweb parameters, we used $C = 0.15, 0.2, 0.25$ and $n_S = 30, 45, 60, 75$. We simulated 6 years of metacommunity dynamics and 50 replicates for each point in the parameter space. Although individual sites are reset every year, for large values of *A* there may be no time at which all sites are inactive simultaneously. This potential coupling between consecutive seasons demands simulating the system for more than one year. Besides, prospective tests determined that a 6 year period was enough to capture the long-term behavior of the system. We define instantaneous species persistence at time *t* as the ratio between species richness at *t* and the number of species in the regional pool. Similarly, we define instantaneous community biomass as the sum of all species’ biomasses at the equilibrium of Eq. (1) We calculate time series for $\alpha$ (average local) and $\gamma$ (regional) persistence, denoted as ${\mathcal {P}}_\alpha (t)$ and ${\mathcal {P}}_\gamma (t)$ respectively.

We average these instantaneous magnitudes over the entire length of the simulation using the continuous power mean (see “Supplementary Information”) to obtain the scalars ${\mathcal {P}}_\alpha$, ${\mathcal {P}}_\gamma$. Following^39^, we calculate beta persistence ${\mathcal {P}}_\beta$ as ${\mathcal {P}}_\gamma /{\mathcal {P}}_\alpha$. Similarly, we calculate the time series for regional metacommunity biomass ${\mathcal {B}}_\gamma (t)$ as the community biomass summed over all sites. Then, we define $\beta$ biomass ${\mathcal {B}}_\beta (t)$ as the coefficient of variation of local community biomasses across sites. Finally, we compute their power means to obtain scalars ${\mathcal {B}}_\gamma$ and ${\mathcal {B}}_\beta$ respectively. There is no need to obtain ${\mathcal {B}}_\alpha$ because it is proportional to ${\mathcal {B}}_\gamma$. The final values extracted from a model run are the variables ${\mathcal {P}}_\alpha$, ${\mathcal {P}}_\gamma$, ${\mathcal {P}}_\beta$, ${\mathcal {B}}_\beta$ and ${\mathcal {B}}_\gamma$. These variables encapsulate fundamental biodiversity patterns in the context of spatially distributed ecological communities.

We focus on assessing how increasing landscape asynchrony *A* affects biodiversity patterns. For a given $x\in \{{\mathcal {P}}_\alpha , {\mathcal {P}}_\gamma , {\mathcal {P}}_\beta , {\mathcal {B}}_\beta , {\mathcal {B}}_\gamma \}$ we define the *sensitivity of x to an increase in A* as

3 $$\begin{aligned} {\mathcal {S}}[x]&= \frac{x_H - x_L}{A_{H} - A_{L}} \end{aligned}$$

where $A_L$ and $A_H$ are referential low and high values for *A*, respectively, and $x_L$ and $x_H$ are the values of *x* for $A_L$ and $A_H$ respectively. In our experiments, we set $A_L=0$ and $A_H=0.5$. For brevity, we write *A*-*sensitivities* to denote the elements of the set $\{{\mathcal {S}}[{\mathcal {P}}_\alpha ]$, ${\mathcal {S}}[{\mathcal {P}}_\beta ]$, ${\mathcal {S}}[{\mathcal {P}}_\gamma ]$, ${\mathcal {S}}[{\mathcal {B}}_\beta ]$, ${\mathcal {S}}[{\mathcal {B}}_\gamma ]\}$ containing the response variables used in our main experiments. We quantified the effects of LSFs versus RSFs on the *A*-sensitivities. As predictor variables, we used *T* and $\lambda$, which regulate local community stability, and ${\hat{a}}=\log _{10}(a)$, which regulates regional processes. We also tested all possible quadratic interactions among the three main predictors. Standardized effect sizes were obtained from the coefficients of multiple linear regressions after rescaling the main predictors to z-scores. Also, we tested the possibility of removing some of the predictors by comparing the corrected Akaike information criterion (AICc) values for all linear models nested within our full model.

## Results

As a starting point, we analyze how LSFs (*T* and $\lambda$) affect species persistence and community biomass as a result of the assembly process of a single community from the regional species pool. For this particular experiment, the assembly process occurs in the context of unlimited access to the species pool, and permanent habitat availability, i.e., the site is always active. Therefore, for this limiting case, community composition is mainly governed by local processes. Also, since ${\mathcal {P}}_\alpha ={\mathcal {P}}_\gamma$ we will denote species persistence as ${\mathcal {P}}$. Similarly, we will use ${\mathcal {B}}$ to represent metacommunity biomass ${\mathcal {B}}_\gamma$.

**Figure 1:**
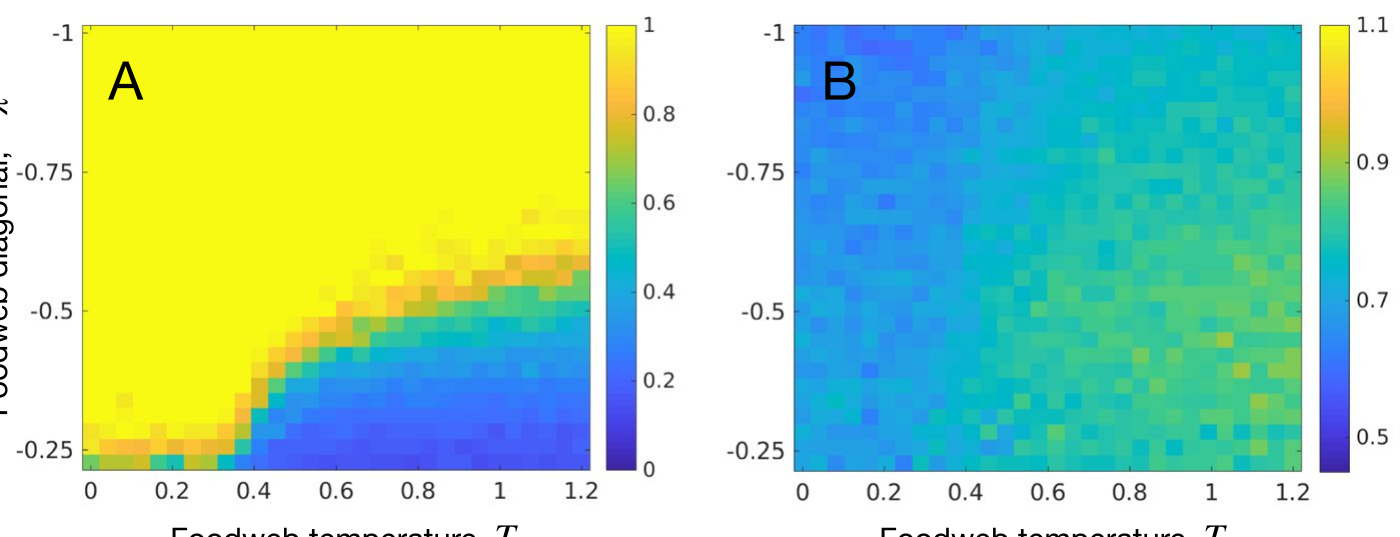
Local community stability. Species persistence (**A**) and $\log _{10}$ community biomass (**B**), in a single local community with colonization from a regional pool, as a function of foodweb diagonal values ($-\lambda$) and foodweb temperature values *T*. Foodweb complexity parameters are $n_S=45$ and $C=0.2$. Each cell value shows the mean of 50 replicates.

An analysis of Fig. 1 reveals that both *T* and $\lambda$ exerted marked effects on community diversity and biomass. Species persistence ${\mathcal {P}}$ decreased with *T* and increased with $\lambda$ (Fig. 1A). The opposite trend was obtained for community biomass ${\mathcal {B}}$ (Fig. 1B). Figs. SI2 and SI3 in “Supplementary Information” show the influence of foodweb complexity ($n_S$ and *C*) on ${\mathcal {P}}$ and ${\mathcal {B}}$. On one hand, $n_S$ lowered ${\mathcal {P}}$ in the sense that it shrank the region in the $\lambda , T$ parameter space where ${\mathcal {P}}$ is high. On the other hand, $n_S$ increased ${\mathcal {B}}$. By contrast, *C* decreased both ${\mathcal {P}}$ and ${\mathcal {B}}$.

Next, we extend the experiments to metacommunities in dynamic landscapes.

To gain initial insights into the effects of landscape asynchrony *A* on metacommunity attributes, we run our full model using two dispersal rates. We assessed the relative changes in ${\mathcal {P}}$ and ${\mathcal {B}}$ while varying *A* from 0 to 0.5

**Figure 2:**
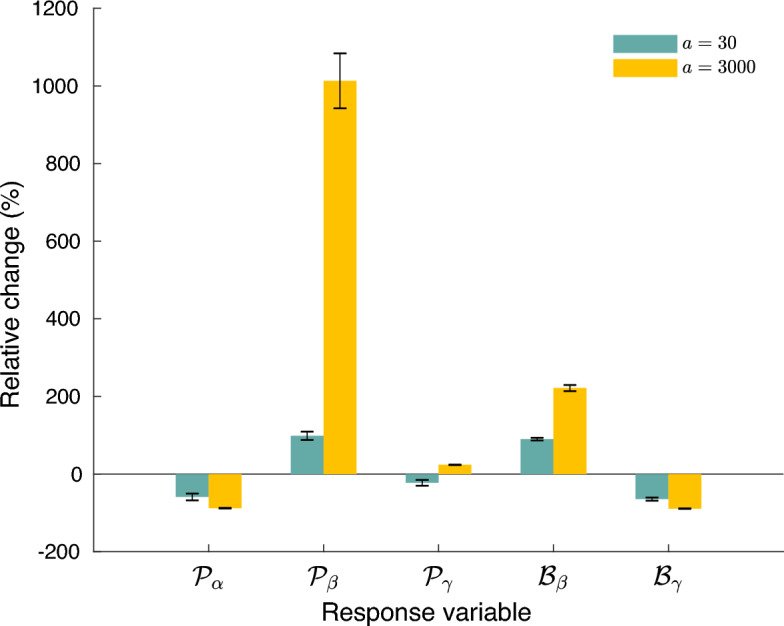
Effects of landscape asynchrony. Relative change of each response variable when changing landscape asynchrony *A* from 0.0 to 0.5 at two values of dispersal ability *a*. Mean and SE over 50 replicates, with $n_S=45$, $C=0.2$, $n_P=100$, $F=50$, $T=0.2$, and $\lambda =0.47$.

As we see in Fig. 2, increasing *A* reduced both ${\mathcal {P}}_\alpha$ and ${\mathcal {B}}_\gamma$, while it increased ${\mathcal {P}}_\beta$ and ${\mathcal {B}}_\beta$. The response of ${\mathcal {P}}_\gamma$ was comparatively smaller and its sign depended on dispersal ability *a*. In general, increasing *a* strengthened the effect of *A*, especially for ${\mathcal {P}}_\beta$.

### Effects of local and regional stabilizing factors

**Figure 3:**
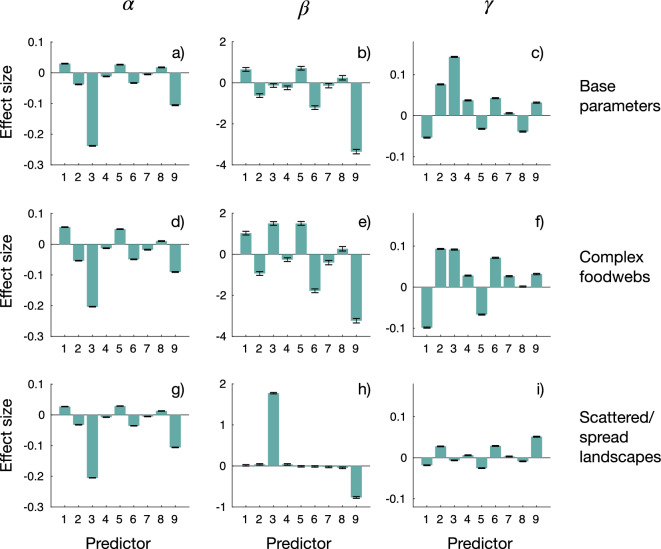
Effect sizes on *A*-sensitivities ${\mathcal {S}}[{\mathcal {P}}_\alpha ]$, ${\mathcal {S}}[{\mathcal {P}}_\beta ]$,and ${\mathcal {S}}[{\mathcal {P}}_\gamma ]$. Predictors are foodweb temperature *T* (1), self limitation $\lambda$ (2), dispersal ability ${\hat{a}}=\log _{10}a$ (3), $T \cdot \lambda$ (4), $T \times {\hat{a}}$ (5), $\lambda \times {\hat{a}}$ (6), $T^2$ (7), $\lambda ^2$ (8), and ${\hat{a}}^2$ (9). For plots a-c, parameters are $n_S=45$ (species richness), $C=0.2$ (connectance), $n_P=50$ (number of sites), $F=50$ (a surrogate for spatial modularity). For plots d-f, $n_S=75$, $C=0.25$, $n_P=50$, $F=50$. For plots g-i, $n_S=45$, $C=0.2$, $n_P=10$, $F=5$.

According to the AICc, the full model outperformed all nested, smaller models. Fig. 3 shows the effect sizes of each predictor on *A*-sensitivities of species persistence.

Dispersal ability ${\hat{a}}$ was a strong predictor of ${\mathcal {S}}[{\mathcal {P}}_\alpha ]$, ${\mathcal {S}}[{\mathcal {P}}_\beta ]$, and ${\mathcal {S}}[{\mathcal {P}}_\gamma ]$. Moreover, ${\hat{a}}$ determined *A*-sensitivities in a nonlinear way, as indicated by the effect sizes of ${\hat{a}}^2$ (Fig. 3). Increasing ${\hat{a}}$ tended to reduce ${\mathcal {S}}[{\mathcal {P}}_\alpha ]$, as indicated by the negative effect sizes. This trend held for metacommunities governed by the three parameter sets: base condition (Fig. 3a), complex foodwebs (Fig. 3d), and scattered/spread landscapes (Fig. 3g). For all the cases in Fig. 3, the effect of ${\hat{a}}$ over ${\mathcal {S}}[{\mathcal {P}}_\beta ]$ was parameter (foodweb and landscape) dependent and highly nonlinear. However, ${\hat{a}}$ was not the strongest predictor of ${\mathcal {S}}[{\mathcal {P}}_\beta ]$ for denser/modular landscapes (Fig. 3b,e). For the base case, ${\hat{a}}$ had a positive and mostly linear effect on ${\mathcal {S}}[{\mathcal {P}}_\gamma ]$ (Fig. 3c). For complex foodwebs, this effect, although qualitatively the same as for the base case, was not the dominant one (Fig. 3f). For scattered/spread landscapes, the effect of ${\hat{a}}$ on ${\mathcal {S}}[{\mathcal {P}}_\gamma ]$, although relatively small, was highly nonlinear (Fig. 3i). Predictors $\lambda$ and *T* exerted noticeable effects on all *A*-sensitivities, except ${\mathcal {S}}[{\mathcal {P}}_\beta ]$ in scattered landscapes. The effect sizes of $\lambda$ on persistence-related *A*-sensitivities were of similar magnitude but, as expected, of opposite sign than those of *T*. The quadratic terms for $\lambda$ and *T* were relatively small. Strengthening local stability, either through increasing $\lambda$ or decreasing *T*, led to a decrease in ${\mathcal {S}}[{\mathcal {P}}_\alpha ]$, and of ${\mathcal {S}}[{\mathcal {P}}_\beta ]$ (except for scattered landscapes), while it increased ${\mathcal {S}}[{\mathcal {P}}_\gamma ]$. Interaction effects between ${\hat{a}}$ and both $\lambda$ and *T* were of considerable size for ${\mathcal {S}}[{\mathcal {P}}_\gamma ]$, and for ${\mathcal {S}}[{\mathcal {P}}_\beta ]$ (base parameters and complex foodwebs). Predictor $T \times {\hat{a}}$ increased ${\mathcal {S}}[{\mathcal {B}}_\beta ]$ while it decreased ${\mathcal {S}}[{\mathcal {B}}_\gamma ]$. Predictor $\lambda \times {\hat{a}}$ had the opposite effects.

### Effects of landscape structure

The number of sites in the landscape $n_P$, and the excess factor *F*, exerted noticeable effects on the relative effect sizes of predictors on *A*-sensitivities. In general, the absolute sizes of the effects increased with $n_P$, except on ${\mathcal {S}}[{\mathcal {B}}_\beta ]$ (Figs. SI4–SI8 in “Supplementary Information”). Increases in *F* tended to decrease the effects exerted by ${\hat{a}}$ and ${\hat{a}}^2$ on ${\mathcal {S}}[{\mathcal {P}}_\alpha ]$. Conversely, for ${\mathcal {S}}[{\mathcal {P}}_\gamma ]$, these effect sizes tended to be magnified by *F*. Large $n_P$ values strengthened the effects of *F* on persistence-related *A*-sensitivities. The sensitivity ${\mathcal {S}}[{\mathcal {B}}_\beta ]$ remained almost fully explained by ${\hat{a}}$ (and ${\hat{a}}^2$) for all the explored parameter space. Interestingly, for ${\mathcal {S}}[{\mathcal {P}}_\gamma ]$ the importance of *T* and $\lambda$ relative to that of ${\hat{a}}$ increased for small values of $n_P$ and *F*.

### Effects of foodweb topology

Foodweb parameters *C* and $n_S$ moderately influenced the relative effect sizes of predictors on the *A*-sensitivities (Figs. SI9–SI13 in “Supplementary Information”). For ${\mathcal {S}}[{\mathcal {P}}_\alpha ]$, as foodweb complexity (*C* and $n_S$) increased, the relative importance of *T* and $\lambda$ increased respect to that of ${\hat{a}}$. This is consistent with our first local stability analysis (Fig. SI2 in “Supplementary Information”), which showed that more complex communities display a smaller stability region on the $\lambda$-*T* plane. Similarly, effects of $\lambda$ and *T* on ${\mathcal {S}}[{\mathcal {P}}_\gamma ]$ increased, relative to the ones of ${\hat{a}}$, as foodwebs were more complex. Nonlinear predictors $T \times {\hat{a}}$ and $\lambda \times {\hat{a}}$ also exhibited stronger effects for complex foodwebs. For ${\mathcal {S}}[{\mathcal {P}}_\beta ]$, increasing the foodweb complexity led to larger effect sizes of the main predictor variables, and those of $T \times {\hat{a}}$ and $\lambda \times {\hat{a}}$. The *A*-sensitivities ${\mathcal {S}}[{\mathcal {B}}_\beta ]$ and ${\mathcal {S}}[{\mathcal {B}}_\gamma ]$ showed minor changes over the foodweb complexity gradient.

## Discussion

Our main results show that increasing asynchrony *A* among site availability periods reduces both local species persistence and biomass, while it raises among-habitat dissimilarity with respect to these metrics. Interestingly, regional persistence increased with *A*, particularly at high dispersal rates, even though regional biomass decreased. The sensitivity of metacommunities to increased landscape asynchrony was determined by both LSFs and RSFs. Roughly speaking, dispersal was the dominant predictor of *A*-sensitivities across a wide array of conditions, although the contribution of LSFs (both through their main effects and interactions) had a considerable influence on the *A*-sensitivity of regional persistence, and among-site dissimilarity in species richness. The importance of LSFs was particularly strong for scattered/spread landscapes and complex foodwebs. Among the previous studies addressing the role of local versus RSFs on metacommunity stability,^17^ and^16^ stand out.^17^ analyze the local asymptotic stability of metacommunities, and show that the probability of a metacommunity being stable increases with the propensity to stability of local communities (governed by species richness, foodweb connectance, mean interaction strength, and self-regulation strength), as well as with dispersal rate. At the same time, they showed that the likelihood of metacommunity stability increases with habitat complexity (number of functionally distinct sites).

Applying a similar approach,^16^ found that the key determinants of local community stability, namely species richness and food web connectance, also play a crucial role in shaping the stability of the entire metacommunity. In essence, more stable local communities tend to contribute to the stability of the metacommunity. Moreover, dispersal can stabilize metacommunities composed of unstable prone (i.e. more complex) communities, potentially reversing the negative complexity-stability relation under certain conditions. Our findings are in line with^17^ and^16^ in that both local community stability and dispersal rates raise both local and regional diversity. Note that these studies rely on Lyapunov stability analysis, which characterizes the system’s response to infinitesimal perturbations from equilibrium. However, a strong Lyapunov stability does not necessarily guarantee system robustness in the face of large perturbations such as those explored in our study where all species at a site become extinct when their habitat transitions from available to unavailable. In the following paragraphs we delve into the mechanisms explaining the responses of metacommunities to asynchrony in habitat availability (hereafter “asynchrony”) and how foodweb and landscape complexity modulate these responses.

We begin by noting that increasing synchrony tends to reduce the average temporal overlap among active sites. This inhibits dispersal, leading to lower local diversity and a larger among-site dissimilarity in species composition. However, landscape asynchrony can have positive effects on regional diversity due to a compensatory effect. This is because a larger asynchrony results in a larger fraction of the year when active sites host species, promoting the prompt colonization of newly activated sites. However, LSFs determine whether asynchrony has a net positive or negative effect on regional biodiversity, as we will elaborate on later.

### Dispersal

The increase in ${\mathcal {S}}[{\mathcal {P}}_\alpha ]$ with dispersal ability, ${\hat{a}}$, can be explained by examining Fig. SI14 in “Supplementary Information”. Due to dispersal limitation, the range of ${\mathcal {P}}_\alpha$ is narrower for slow dispersal. Thus, a slow dispersal results in small ${\mathcal {P}}_\alpha$, which cannot be significantly reduced by increasing landscape asynchrony. This leads to a small ${\mathcal {S}}[{\mathcal {P}}_\alpha ]$. In contrast, a faster dispersal leads to higher levels of ${\mathcal {P}}_\alpha$ that can be decreased readily by increments in asynchrony, yielding a negative ${\mathcal {S}}[{\mathcal {P}}_\alpha ]$. These cases explain the negative effects of dispersal ability on ${\mathcal {S}}[{\mathcal {P}}_\alpha ]$ in Fig. 3 a, d, and g.

For dense landscapes and slow dispersal, ${\mathcal {P}}_\gamma$ decreased only slightly with asynchrony because having a few species-rich communities yields a high ${\mathcal {P}}_\gamma$. An intermediate dispersal level prevents reductions in ${\mathcal {P}}_\gamma$. For high dispersal levels, asynchrony increases ${\mathcal {P}}_\gamma$ in many cases, particularly for metacommunities exhibiting high ${\mathcal {P}}_\gamma$ when sites are perfectly synchronized. This can be explained by the compensatory effect described earlier. These cases elucidate the effects of dispersal on ${\mathcal {S}}[{\mathcal {P}}_\gamma ]$ as shown in Figs. 3 c, and f. A similar response has been observed in models of competitive metacommunities and, more recently, in multitrophic metacommunities^40^, where a positive relationship exists between spatially uncorrelated environmental fluctuations and stability. In the cases of scattered/spread landscapes, the effect of dispersal ability on ${\mathcal {S}}[{\mathcal {P}}_\gamma ]$ was markedly nonlinear. For slow dispersal, ${\mathcal {S}}[{\mathcal {P}}_\gamma ]\approx 0$ since, regardless of asynchrony, ${\mathcal {P}}_\gamma \approx 0$. Increasing dispersal ability raises ${\mathcal {P}}_\gamma$ when sites are synchronized. A higher asynchrony pushes down ${\mathcal {P}}_\gamma$, resulting in a negative ${\mathcal {S}}[{\mathcal {P}}_\gamma ]$. When dispersal is fast, ${\mathcal {P}}_\gamma$ remains relatively high, and there is a positive ${\mathcal {S}}[{\mathcal {P}}_\gamma ]$ because of the compensatory effect. The change in the sign of ${\mathcal {S}}[{\mathcal {P}}_\gamma ]$ leads to the nonlinear effect of dispersal in Fig. 3i.

Dispersal affects ${\mathcal {S}}[{\mathcal {P}}_\beta ]$ nonlinearly for dense landscapes (Figs. 3b, e), reaching a maximum at intermediate dispersal rates and yielding small values for both small and large dispersal. When dispersal is slow, most sites are unpopulated and, therefore homogeneous, regardless of asynchrony. For fast dispersal, sites reachable from the mainland are homogeneously populated. Increasing asynchrony reduces the number of available sites and therefore ${\mathcal {P}}_\beta$ increases moderately. In contrast, intermediate dispersal values, induces dissimilarity in species composition across space. Besides, landscape asynchrony reduces the number of available sites, leading to an even greater increase in ${\mathcal {P}}_\beta$. This yields a marked positive ${\mathcal {S}}[{\mathcal {P}}_\beta ]$.

The described effects of dispersal ability on ${\mathcal {S}}[{\mathcal {P}}_\beta ]$, along with the underlying mechanisms, also apply to scattered/spread landscapes. Here, landscape asynchrony increases ${\mathcal {P}}_\beta$ at high dispersal ability. The increased average distance among sites in scattered/spread landscapes lowers effective rates of dispersal, potentially weakening its homogenizing effect.

The patterns of metacommunity *A*-sensitivity of biomass can be explained using similar arguments as those stated before. Basically, dispersal ability fosters biomass abundance, and homogenization across sites. Therefore, dispersal has a positive effect on ${\mathcal {S}}[{\mathcal {B}}_\beta ]$ and a negative effect on ${\mathcal {S}}[{\mathcal {B}}_\gamma ]$ (see Figs. 2, SI7, SI8, SI12 and SI13 in “Supplementary Information”).

Our results align with earlier studies that emphasize the critical role of dispersal as a driving force behind metacommunity dynamics and resultant diversity patterns. The influential work of^41,42^, assuming a spatially implicit patch dynamics archetype for competitive metacommunities, posited that dispersal leads to a humped response in $\alpha$-diversity, accompanied by decreasing trends in both $\beta$-diversity and $\gamma$-diversity. Recently,^19^ adopted a more comprehensive approach encompassing a wider range of archetypes and identified similar trends to those in^41,42^, albeit with qualitative differences for some settings. Presently, research examining the impacts of dispersal on multitrophic metacommunities^43^, e.g. yields results analogous to those observed in competitive metacommunities.

However, these theoretical predictions often diverge from empirical results^44^. In our metacommunity assembly model, we obtained positive responses of ${\mathcal {P}}_\alpha$ and ${\mathcal {P}}_\gamma$, and a negative response of ${\mathcal {P}}_\beta$ to increases in dispersal ability. When considering landscapes subjected to perturbations, dispersal can mitigate their impact on local populations by subsidizing populations from undisturbed sites^45^. However, in multitrophic metacommunities, the ability of dispersal to maintain local populations differs among trophic groups^46,47^. In our study, we found that dispersal: a) magnifies the negative effect of asynchrony on ${\mathcal {P}}_\alpha$. b) magnifies the positive effect of asynchrony on ${\mathcal {P}}_\beta$ over a large region of the parameter space. c) shifts the effect of asynchrony on ${\mathcal {P}}_\gamma$ from negative to positive.

### Local stabilizing factors

Explaining why LSFs (predictors *T* and $\lambda$) have a significant effect on metacommunity sensitivity, especially on ${\mathcal {S}}[{\mathcal {P}}_\gamma ]$, is straightforward when examining Figs. SI14 and SI15 in “Supplementary Information”. Note that for large values of *a*, regardless of asynchrony and $n_P$, stable-prone foodwebs tend to produce metacommunities with high regional persistences. We observe a similar trend for ${\mathcal {S}}[{\mathcal {P}}_\alpha ]$ when sites are synchronized. We illustrate the processes behind these trends by analyzing metacommunity dynamics on idealized star-shaped landscapes (see “Supplementary Information”, Section Star experiment).

This experiment reveals that all effects of LSFs on *A*-sensitivities can be explained by the interplay among the time-averaged values of three variables: the number of available sites, the number of species per site, and the dissimilarity of species composition among sites. Regardless of local stability-proneness, a reduction in available sites by increasing asynchrony drives down ${\mathcal {P}}_\alpha$. While for stable-prone foodwebs all available sites hosted essentially all species, for unstable-prone foodwebs, available sites hosted a small fraction of the species pool, which leads to a decrease in ${\mathcal {P}}_\alpha$. In contrast to stable-prone foodwebs, unstable-prone foodwebs induce a high among-site heterogeneity in species composition. This, combined with the reduction in available sites, results in a decrease in ${\mathcal {P}}_\gamma$. This yields relatively high ${\mathcal {P}}_\gamma$ values in spite of the low ${\mathcal {P}}_\alpha$.

From previous considerations, for unstable-prone communities, ${\mathcal {P}}_\alpha$ maintains low values regardless of asynchrony resulting in a small ${\mathcal {S}}[{\mathcal {P}}_\alpha ]$. For stable-prone communities, ${\mathcal {P}}_\alpha$ is high for $A=0$ and low for high asynchrony values. Thus, ${\mathcal {S}}[{\mathcal {P}}_\alpha ]$ becomes very negative. Hence ${\mathcal {S}}[{\mathcal {P}}_\alpha ]$ decreases with local stability-proneness. Similarly, for unstable-prone communities ${\mathcal {P}}_\gamma$ decreases as asynchrony increases, resulting in a negative ${\mathcal {S}}[{\mathcal {P}}_\gamma ]$. For stable-prone communities, ${\mathcal {P}}_\gamma$ remains insensitive to changes in asynchrony. Therefore, ${\mathcal {S}}[{\mathcal {P}}_\gamma ]$ increases with local stability-proneness. The combination of few species per site and limited available sites leads to a very small ${\mathcal {P}}_\alpha$ for unstable-prone communities and high values of asynchrony. Also, ${\mathcal {P}}_\gamma$ is relatively high, resulting in a large ${\mathcal {P}}_\beta$. This explains the negative effects of local stability-proneness on ${\mathcal {S}}[{\mathcal {P}}_\beta ]$. In the case of scattered landscapes, the effects of *T* and $\lambda$ on ${\mathcal {S}}[{\mathcal {P}}_\beta ]$ were negligible. This is due to the longer routes for dispersal (i.e. longer distances between adjacent sites), which makes dispersal rate outweigh all the other predictors.

Spatial heterogeneity in community biomass is also altered by LSFs. Specifically, ${\mathcal {B}}_\gamma$ decreases with asynchrony, although local stability proneness buffers this reduction in metacommunity biomass. LSFs do not exert any noticeable effect on ${\mathcal {S}}[{\mathcal {B}}_\beta ]$.

### Landscape structure

There has been a growing acknowledgment of the importance of landscape structure on metacommunity diversity^48–51^. Moreover, environmental changes alter landscape structure at different temporal and spatial scales^30^. Earlier spatially implicit metapopulation models show that species persistence is highly sensitive to landscape dynamics^52,53^. In a metacommunity context, using an experimental system of only two sites,^45^ showed that local population densities and species persistence exhibit different responses to periodic perturbations on local sites and that dispersal capabilities play a key role in the recovery of species after perturbations. To understand and project the consequences of these changes on biodiversity it is natural to resort to an approach based on spatially explicit, dynamic landscapes. More recent advances using a neutral spatially explicit model, highlight that temporal changes in landscape structure may shape metacommunity biodiversity, showing that fluctuating landscape connectivity may enhance local and regional diversity, relative to static landscapes with constant connectivity^54^. In this study, we explore the behavior of multitrophic metacommunities in spatially explicit dynamic landscapes.

The most noticeable effects of landscape structure are related to the number of sites and its influence on $\beta$ and $\gamma$ diversity, and metacommunity biomass. Landscapes with more sites imply shorter distances between sites, resulting in faster dispersal. This leads to a positive effect of $n_P$ on ${\mathcal {P}}_\gamma$. A similar effect is observed for *F* on ${\mathcal {P}}_\gamma$ because of the short distances between the mainland and each site in its module. Regardless of landscape structure, asynchrony reduces the availability of dispersal routes among sites by drastically reducing (or even canceling) the time intervals during which dispersal between sites is possible. The effect of *a* on ${\mathcal {S}}[{\mathcal {P}}_\gamma ]$ is stronger on denser and more modular landscapes. This is due to the combination of the positive effect of *a*, described earlier, the positive effects of $n_P$ and *F* on dispersal rates, and $n_P$ increasing the probability of having (asynchronously) activated sites present throughout the season. The relationship between $n_P$ and *F* and dispersal rates also helps explain the observed patterns of variation of ${\mathcal {S}}[{\mathcal {P}}_\beta ]$ across landscape structure parameters. The number of sites $n_P$ strengthens the effect of *a* on ${\mathcal {S}}[{\mathcal {B}}_{\gamma }]$ because regardless of asynchrony, when *a* is very small, ${\mathcal {B}}_{\gamma }\approx 0$ due to dispersal limitations, and ${\mathcal {S}}[{\mathcal {B}}_{\gamma }]\approx 0$. For large values of *a*, and perfectly synchronized sites, we have optimal dispersal conditions, and metacommunity biomass is proportional to $n_P$. Also, ${\mathcal {B}}_{\gamma }(A=1/2)\ll {\mathcal {B}}_{\gamma }(A=0)$ because of a low site occupancy for highly asynchronous landscape dynamics, regardless of $n_P$. It follows that the effect size of *a* grows with $n_P$. Then, the negative effect of landscape asynchrony on metacommunity biomass rises with *a* and $n_P$.

It follows that the effect size of *a* increases with $n_P$, and consequently, the negative impact of landscape asynchrony on metacommunity biomass is amplified with higher values of both *a* and $n_P$.”

The effect of LSFs on *A*-sensitivities increases with both landscape density and modularity. In denser and more modular landscapes, there are many sites near the mainland. In this scenario ${\mathcal {P}}_\gamma$ decreases with asynchrony among sites hosting unstable-prone communities, due to a lower colonization success that leads to fewer species-rich sites. Conversely, with stable-prone communities, colonization is more successful, leading to a higher fraction of rich communities near the mainland. This process, added to the temporal aggregation effect, increases ${\mathcal {P}}_\gamma$ with higher landscape asynchrony. However, these processes do not occur in scattered/spread landscapes, where longer site-mainland distances strongly limit dispersal and prevent increasing ${\mathcal {P}}_\gamma$. The same mechanisms also contribute to explaining the variation in ${\mathcal {B}}_\gamma$ with different landscape structures.

### Foodweb structure

Unlike^55^, we did not find clear evidence of a positive relation between foodweb complexity and regional species persistence. This is not surprising, given the several differences between our model (explicit landscape with nonrandom structure, equilibrium dynamics of species) and theirs (implicit and fully connected landscape, patch dynamics). This issue deserves further study. Our results indicate that the more complex foodwebs are, the larger the effects of LSFs on *A*-sensitivities of ${\mathcal {P}}_\alpha$, ${\mathcal {P}}_\beta$, and ${\mathcal {P}}_\gamma$. The negative effects of $n_S$, and *C* on local community stability proneness are similar to those of *T* and $-\lambda$. The high importance of LSFs exhibited by complex foodwebs can be explained by the high values of $P_\beta$ reached when communities are unstable-prone. The operating mechanism behind the positive effects of LSFs on ${\mathcal {S}}[{\mathcal {P}}_\gamma ]$ was explained using the star experiment (see “Supplementary Information” Section Star experiment). This effect vanishes for inherently stable foodwebs, such as those with low $n_S$ and *C*, because LSFs are less critical in determining equilibrium population sizes, extinction probability, and receptivity to immigrants. Using similar arguments we can explain why the effect sizes of LSFs on ${\mathcal {S}}[{\mathcal {P}}_\alpha ]$ and ${\mathcal {S}}[{\mathcal {P}}_\beta ]$ increase with the foodweb complexity.

### Final remarks

Our model is based on a random matrix approach ^1,3^ for local communities connected by stochastic dispersal over an explicit random dynamic landscape. This modeling strategy differs from the more frequently used approaches for representing multitrophic metacommunities, as reviewed in^56^. Avoiding numerical integration by focusing on species equilibria, as opposed to transient behavior, allows us to simulate multitrophic metacommunities with many species and many sites efficiently. Our use of a continuous-time Markov chain provides a straightforward means of representing migration events and simulating landscape dynamics with stochastic asynchrony. Finally, our model allows for the coupling of site (de)activation and migration events with changes in local species biomasses via recalculating equilibria at the arrival sites. For future research, our model can be easily extended to incorporate other relevant processes. For instance, it would be worth introducing heterogeneity in site quality^13,43,47^, and dispersal gradients governed by physical^57^ or ecological^53^ conditions.

Previous studies^1,3^ provided valuable insights into isolated community responses to small acute perturbations of species’ abundances by analyzing the Lyapunov stability of linearized systems. However, the role of local stability in a metacommunity context is still not well understood, especially in fluctuating environments. Here, we integrate the roles of local community stability and of connectivity among communities driven by dispersal, in shaping the responses of trophic metacommunities to quasiperiodic habitat creation and destruction. We also present mechanisms that explain how landscape and foodweb complexity determine the relative importance of local versus regional stabilizing factors in maintaining biodiversity patterns in dynamic landscapes. Our findings hold particular relevance in light of the high and growing prevalence of temporary ecosystems, especially aquatic ones^58–61^. Human activities, both directly and indirectly, alter the dynamics of temporary aquatic systems, potentially causing adverse consequences for biodiversity. The comprehension of the dynamics in temporary freshwater systems trails that of their permanent counterparts, hindering the establishment of a robust theoretical framework for devising conservation strategies for these endangered ecosystems^62^.

Our contributions add to the theory of trophic metacommunities and the field of ecological networks on dynamic landscapes^30,63,64^, which require further development in view of the current environmental concerns. However, it would be worthwhile to compare our main results against the outcomes of models that track the full dynamics of the system, as our findings may not necessarily apply to systems with prolonged local transient behaviors. The separation of temporal scales between local and regional processes utilized here could potentially impact the relative importances of LSFs and RSFs in determining metacommunity sensitivity to landscape asynchrony. For instance, the arrival times of successive incoming dispersers might cause temporary interactions among incoming populations, as well as between them and resident species. These interactions, facilitated by a transient phase, could potentially alter the trajectories of local biomasses, leading to alternative responses to activation/deactivation of sites. As a promising avenue for future research, we envision expanding our model to delve into the complex interactions between landscape dynamics and the evolutionary and behavioral adaptations to shifts in abiotic conditions and biotic interactions. Possible extensions include landscape dynamics incorporating habitat loss, for example through site removal or changing site quality. In the context of temporary ponds, this is equivalent to ponds drying out and remaining unfilled. The elimination of existing ponds could result in longer distances among sites, affecting dispersal rates^27^. Another feature to consider is the three-dimensional nature of landscapes (particularly pondscapes), which favors dispersal downwards. This can be translated into asymmetric dispersal rates between sites. Species inhabiting temporary systems face selective pressures that drive the development of adaptations to cope with temporal drought. The development of resistance adaptations, such as relying on resting stages, results in a differentiation between active and passive dispersal, as well as between intra-site and inter-site recolonization. These distinctions may result in different metacommunity dynamics^33^. Finally, attaining a realistic allometric parameterization of dispersal rates among ponds poses a challenge, given that active and passive dispersers may adhere to qualitatively distinct rules. This is because smaller propagules may disperse over longer distances, whereas smaller adult dispersers tend to exhibit the opposite pattern. Overall, there exists a broad spectrum of research opportunities within the realm of metacommunity dynamics in dynamic landscapes. The advancement of this field could accelerate significantly through a tighter integration between theoretical and empirical research endeavors^65,66^.

### Supplementary Information

Supplementary Information.

## Data Availability

No new data were used in this study. Computer codes for simulations are available at https://doi.org/10.5281/zenodo.10014193.
