# Supplementary material for: Both local stability and dispersal contribute to metacommunity sensitivity to asynchronous habitat availability
Source: Sci Rep. 2024 Mar 15;14:6273. doi: 10.1038/s41598-024-56632-y (PMC10943024; doi:10.1038/s41598-024-56632-y)
Supplement: Supplementary file 1 — Supplementary Information. [file 41598_2024_56632_MOESM1_ESM.pdf]

Supplementary Information
for

Both local stability and dispersal contribute to metacommunity sensitivity to asynchronous habitat availability

Pablo Moisset de Espanés & Rodrigo Ramos-Jiliberto

Corresponding author: Rodrigo Ramos-Jiliberto. GEMA Center for Genomics, Ecology & Environment, Universidad Mayor, Chile. E-mail: rodrigo.ramos@umayor.cl

1 *SUPPLEMENTARY METHODS*

1.1 Modular landscape generation algorithm

```
function modular_landscape(n_P, n_E, F, n_C, x)
   // Choose potential locations for sites.
   // Locations are points on the real (x,y) plane.
   Let L be an array of ⌊n_S · F⌋ points in a 512 × 512 square randomly drawn
      from a uniform distribution.

   // Select the first n_C of these locations as cluster centers
   Let C = {1, 2, ..., n_C}.

   // Compute all pairwise distances, and the distances to the closest center
   Let d_{i,j} be the euclidean distance between points L_i and L_j
   Let δ_i = min_{j∈C} d(i, j)

   // Randomly select the non-center sites and combine with centers, yielding
   // the final set of sites S.
   Let V be a set of n_S − n_C integers from {n_C + 1, n_C + 2, ..., ⌊n_S · F⌋} chosen with
      probabilities inversely proportional to δ_i
   Let P = C ∪ V // These vertices will be the landscape sites

   // Compute a minimum spanning tree of the clique of all final sites
   Let K = (P, P × P) // A complete graph with all the vertices
   Let Tree = MST(K) using distances d_{i,j}
   Let E_T = edges(Tree)

   // Randomly choose the remaining edges
```

```
    Let $E$ be a set of $n_E - n_P + 1$, randomly chosen undirected edges from $P \times P - E_T$
        The probability to choose $(i,j)$ is inversely proportional to $d^x_{i,j}$

    Return $G = (P, E + E_T)$ and the weights $d_{i,j}$ for all the edges in $G$
end
```

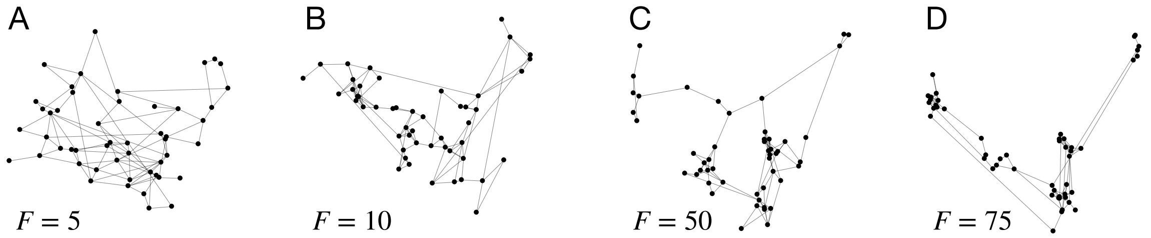

Fig. SI1. Examples of landscapes with different degrees of spatial modularity generated by our algorithm through varying excess factor F. We used 50 sites, 100 edges, 5 modules, and a distance exponent of 2.

1.2 Metacommunity simulation algorithm

There are three types of events:

1. Site activation events, described as triples $\langle t, p, \text{`}\textit{activate}\text{'}\rangle$, meaning at time t site p becomes active.

2. Site deactivation events, described as triples $\langle t, p, \text{`}\textit{deactivate}\text{'}\rangle$, meaning at time t site p becomes inactive.

3. Dispersal events, described as triples $\langle t, s, \textit{dest}\rangle$, meaning at time t individuals of species s move to site *dest*.

The algorithm begins by initializing the event queue with site activation/deactivation events. Let $\mathcal{S}$ be the set of all species and let $\mathcal{P}$ be the set of all landscape sites. For every site p, define the pulse train function $f_p(t) = 1$ if $t \in ACTIVE_p$ and zero otherwise. Let $t_{p,k}^{on}$ be the rising time of the k-th pulse in f_p. Event $\langle t_{p,k}^{on}, p, \text{`}\textit{activate}\text{'}\rangle$ is added to the queue. Similarly, define $t_{p,k}^{off}$ as the falling time of the $k$-th pulse in $f_p$ and add $\langle t_{p,k}^{off}, p, \text{`}\textit{deactivate}\text{'}\rangle$ to the queue.

Conceptually, the algorithm to simulate the metacommunity dynamics is as follows:

```
Init queue Q with site activation/deactivation events

// Set initial state of the systems
for all sites p
   // C[p] is the set of species present at site p
   C[p] = S if p is the mainland
   C[p] = {} if p is insular
   A[p] = 'inactive' // A[p] is the state ('active' or 'inactive') of site p
end


time = 0

while Q ≠ {} //Main simulation loop
   ⟨t_m, s, dest⟩ = next_migration_event()
   ⟨t, p, type⟩ = earliest(Q)
   if t < t_m
      if type='activate'
         A[p] = 'active'
      else
         C[p] = {}
         A[p] = 'inactive'
      end
      Q = Q − ⟨t, p, type⟩
      time = t
   else
      migration_process(s, dest)
      time = t_m
   end
```

```
end

function next_migration_event()
    C1 = {⟨s, dest, orig⟩ such that
            s ∉ C[dest]
            s ∈ C[orig]
            A[dest] = 'active'
            s is basal or preys(s)∩C[dest] ≠ {}}
    C2 = {⟨τ, s, dest, orig⟩ such that
            ⟨s, dest, orig⟩ ∈ C1 and
            τ ~ exp(distance(orig, dest)/a))}
     ⟨τ, s, dest, orig⟩ = earliest(C2)
     return ⟨τ + time, s, dest⟩
end

function migration_process(s, dest)
    C[dest] = C[dest] ∪ {s}
    repeat
        solve
            r_i + ∑_j CM_ij x_j = 0 for i, j ∈ C[dest]
        went_extinct = {s'|x_s' < 0.001}
        C[dest] = C[dest] − went_extinct
    until went_extinct is empty
end
```

1.3 Power mean

Let ρ be a real parameter, let t_0 and t_1 be reals and let $y(t)$ be an integrable function in (t_0, t_1). The average value of y on (t_0, t_1) is

$$\left(\frac{1}{t_1 - t_0}\int_{t_0}^{t_1} y(t)^\rho dt\right)^{\frac{1}{\rho}}. \tag{SI1}$$

This reduces to the arithmetic mean when $\rho = 1$ and is biased toward the time series maxima for larger values of ρ. We chose $\rho = 4$ to reduce the effect of zero abundances during the "inactive" periods. However, our results were robust to changes in ρ values. In our case, $t_0 = 0$, and t_1 is the end of the simulation.

2 ADDITIONAL RESULTS

2.1 Local species persistence and biomass in single communities

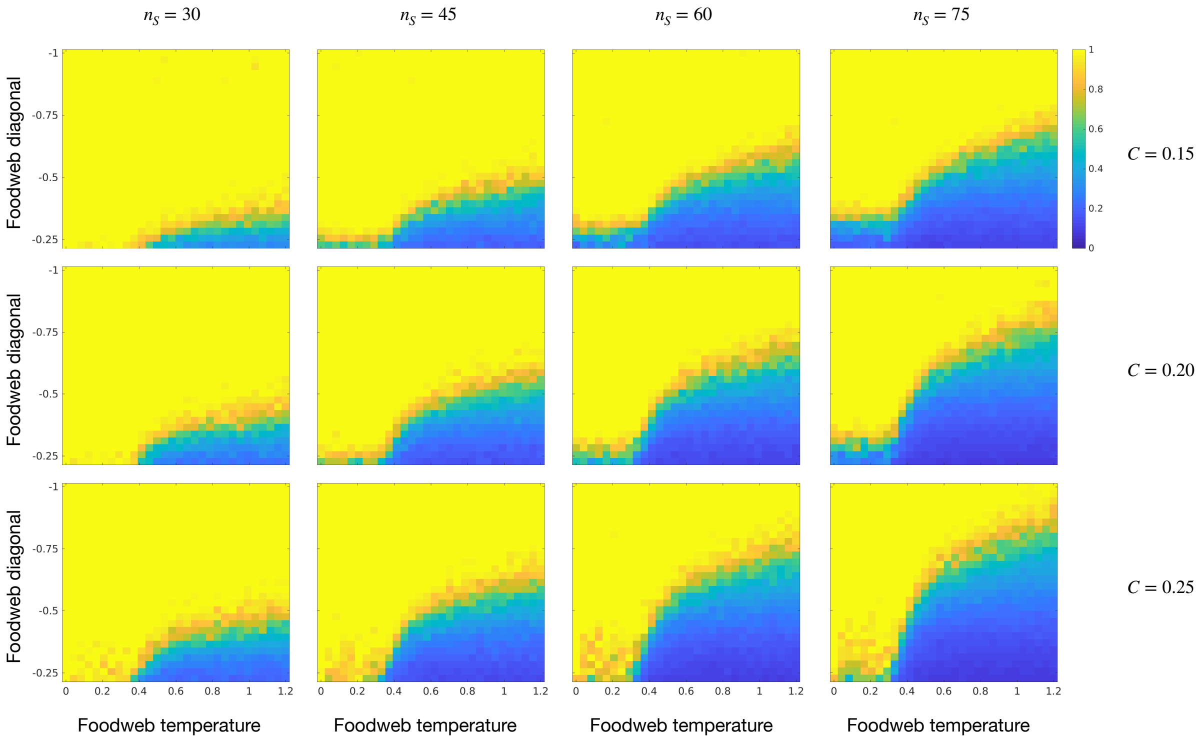

Fig. SI2. Species persistence $\mathcal{P}$ in a single local community with colonization from a regional pool, as a function of foodweb diagonal values (opposite of intensity of self-regulation) and foodweb temperature values (inverse of trophic coherence) for a gradient of richness n_S and connectance C of the species pool. Each cell value shows the mean of 50 replicates.

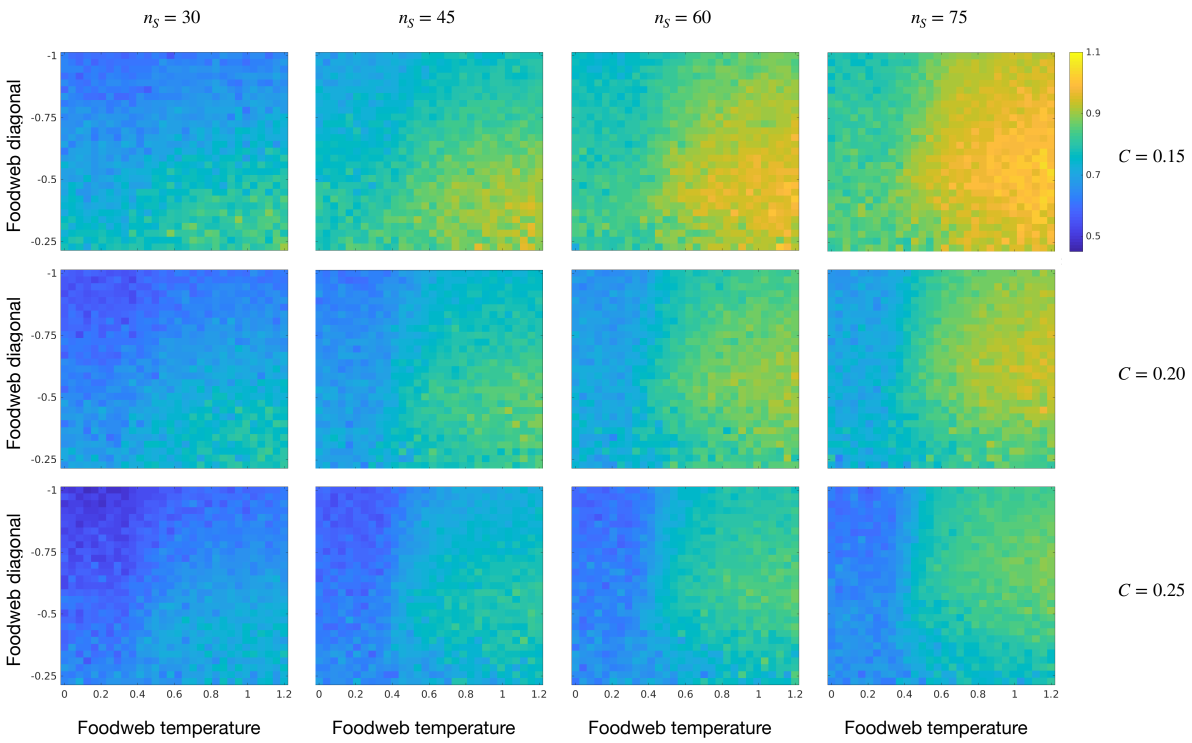

Fig. SI3. Species biomass $\mathcal{B}$ (in $\log_{10}$) in a single local community with colonization from a regional pool, as a function of foodweb diagonal values (opposite of intensity of self-regulation) and foodweb temperature values (inverse of trophic coherence) for a gradient of richness n_S and connectance C of the species pool. Each cell value shows the mean of 50 replicates.

2.2 Effects of local and regional stabilizing factors depending on landscape structure

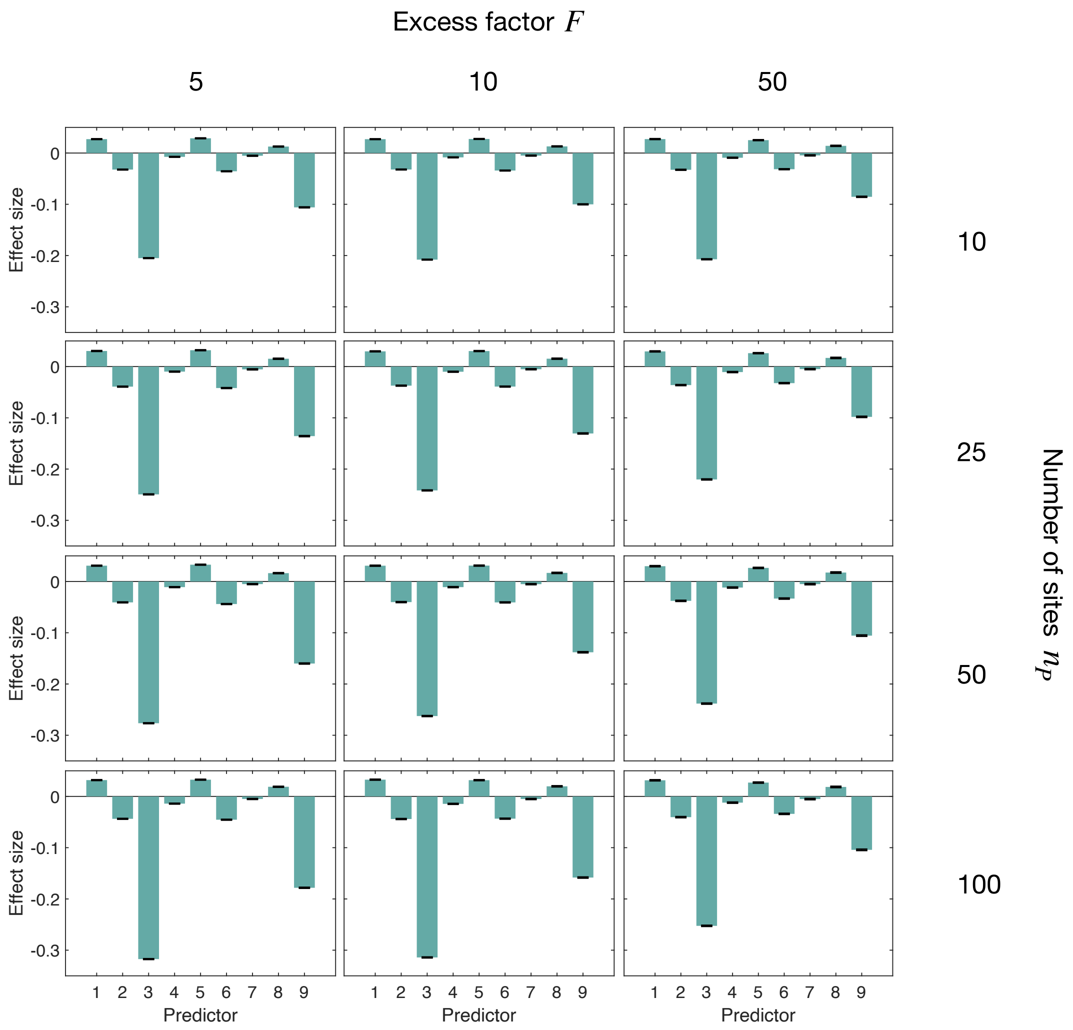

Fig. SI4. Effect sizes on A-sensitivities of local species persistence $\mathcal{S}[\mathcal{P}_\alpha]$ in a gradient of number of sites n_P and excess factor F. Predictors are foodweb temperature T (1), self limitation λ (2), dispersal ability $\hat{a} = \log_{10} a$ (3), $T \cdot \lambda$ (4), $T \times \hat{a}$ (5), $\lambda \times \hat{a}$ (6), T^2 (7), λ^2 (8), and $\hat{a}^2$ (9). Foodweb parameters are $n_S = 45$ and $C = 0.2$

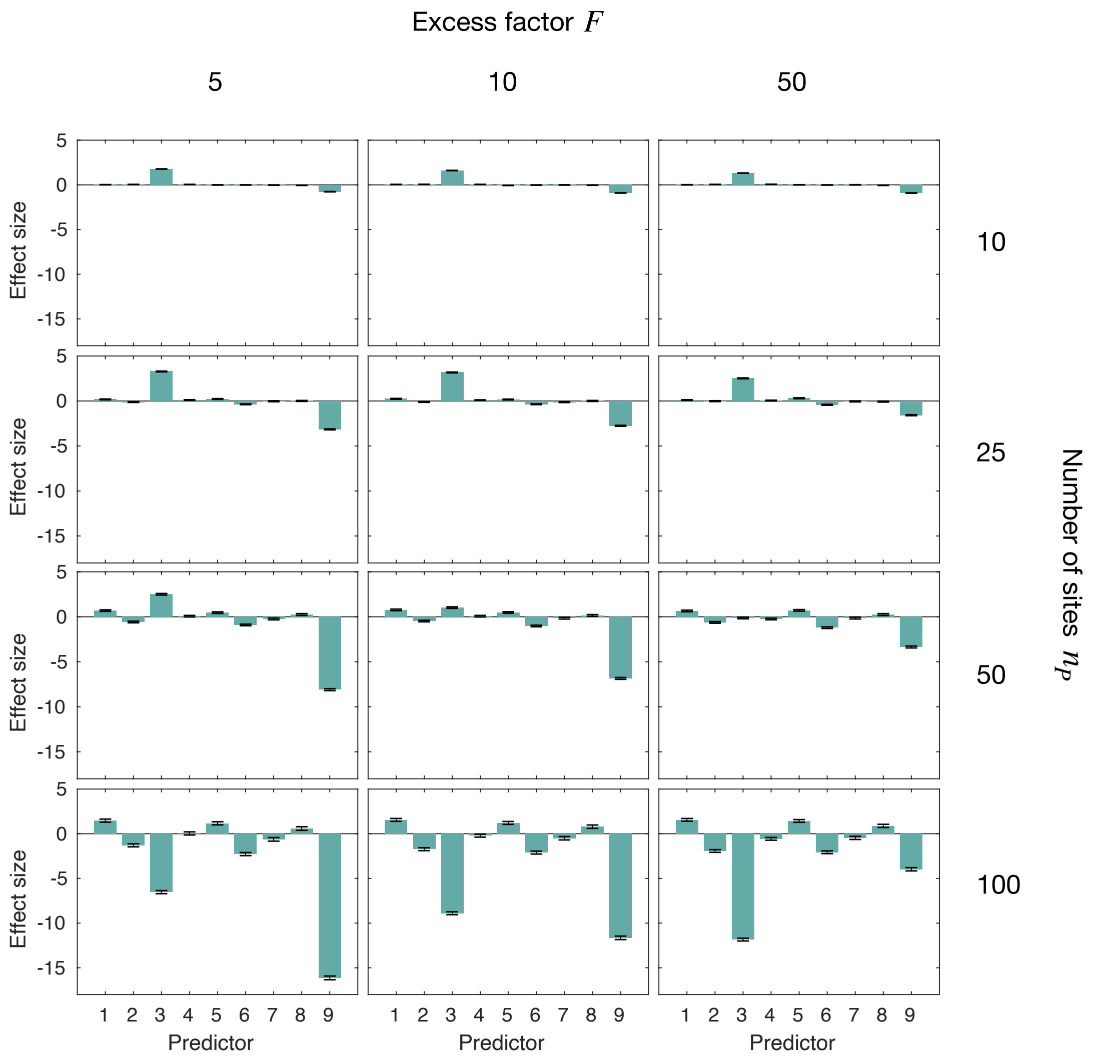

Fig. SI5. Effect sizes on A-sensitivities of among-site dissimilarity in species persistence $\mathcal{S}[\mathcal{P}_\beta]$ in a gradient of number of sites n_P and excess factor F. Predictors are foodweb temperature T (1), self limitation λ (2), dispersal ability $\hat{a} = \log_{10} a$ (3), $T \cdot \lambda$ (4), $T \times \hat{a}$ (5), $\lambda \times \hat{a}$ (6), T^2 (7), λ^2 (8), and $\hat{a}^2$ (9). Foodweb parameters are $n_S = 45$ and $C = 0.2$

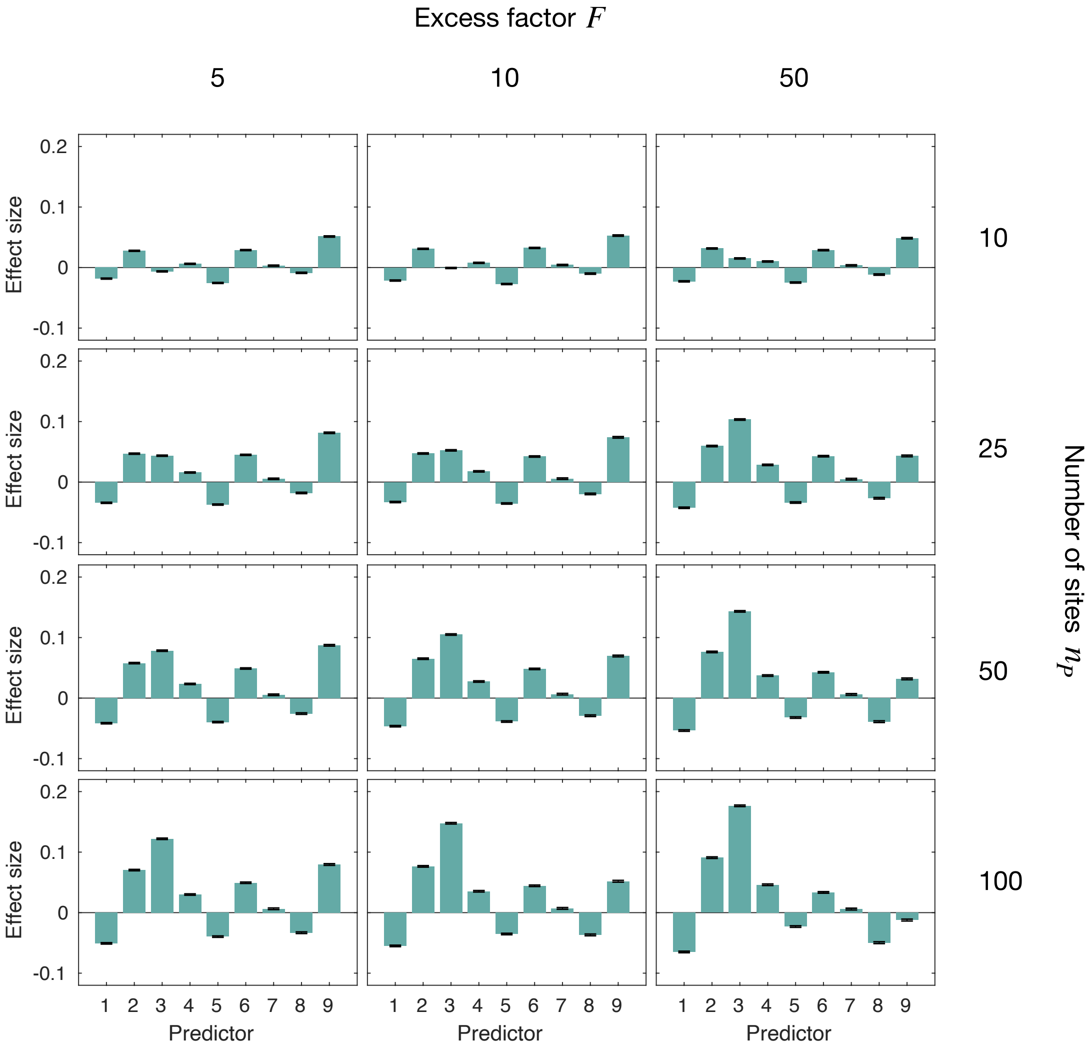

Fig. SI6. Effect sizes on A-sensitivities of regional species persistence $\mathcal{S}[\mathcal{P}_\gamma]$ in a gradient of number of sites n_P and excess factor F. Predictors are foodweb temperature T (1), self limitation λ (2), dispersal ability $\hat{a} = \log_{10} a$ (3), $T \cdot \lambda$ (4), $T \times \hat{a}$ (5), $\lambda \times \hat{a}$ (6), T^2 (7), λ^2 (8), and $\hat{a}^2$ (9). Foodweb parameters are $n_S = 45$ and $C = 0.2$

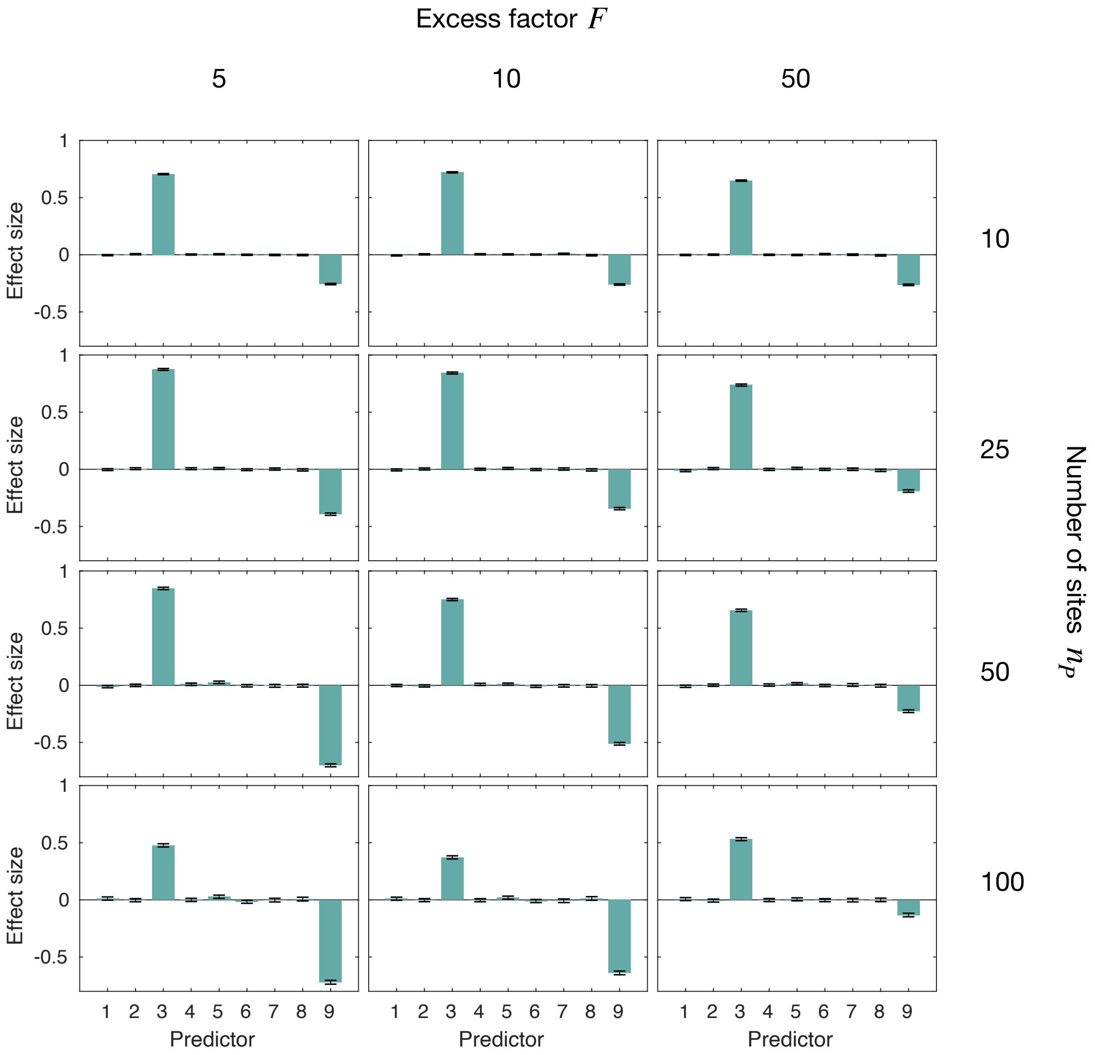

Fig. SI7. Effect sizes on A-sensitivities of among-site dissimilarity in community biomass $\mathcal{S}[\mathcal{B}_\beta]$ in a gradient of number of sites n_P and excess factor F. Predictors are foodweb temperature T (1), self limitation λ (2), dispersal ability $\hat{a} = \log_{10} a$ (3), $T \cdot \lambda$ (4), $T \times \hat{a}$ (5), $\lambda \times \hat{a}$ (6), T^2 (7), λ^2 (8), and $\hat{a}^2$ (9). Foodweb parameters are $n_S = 45$ and $C = 0.2$

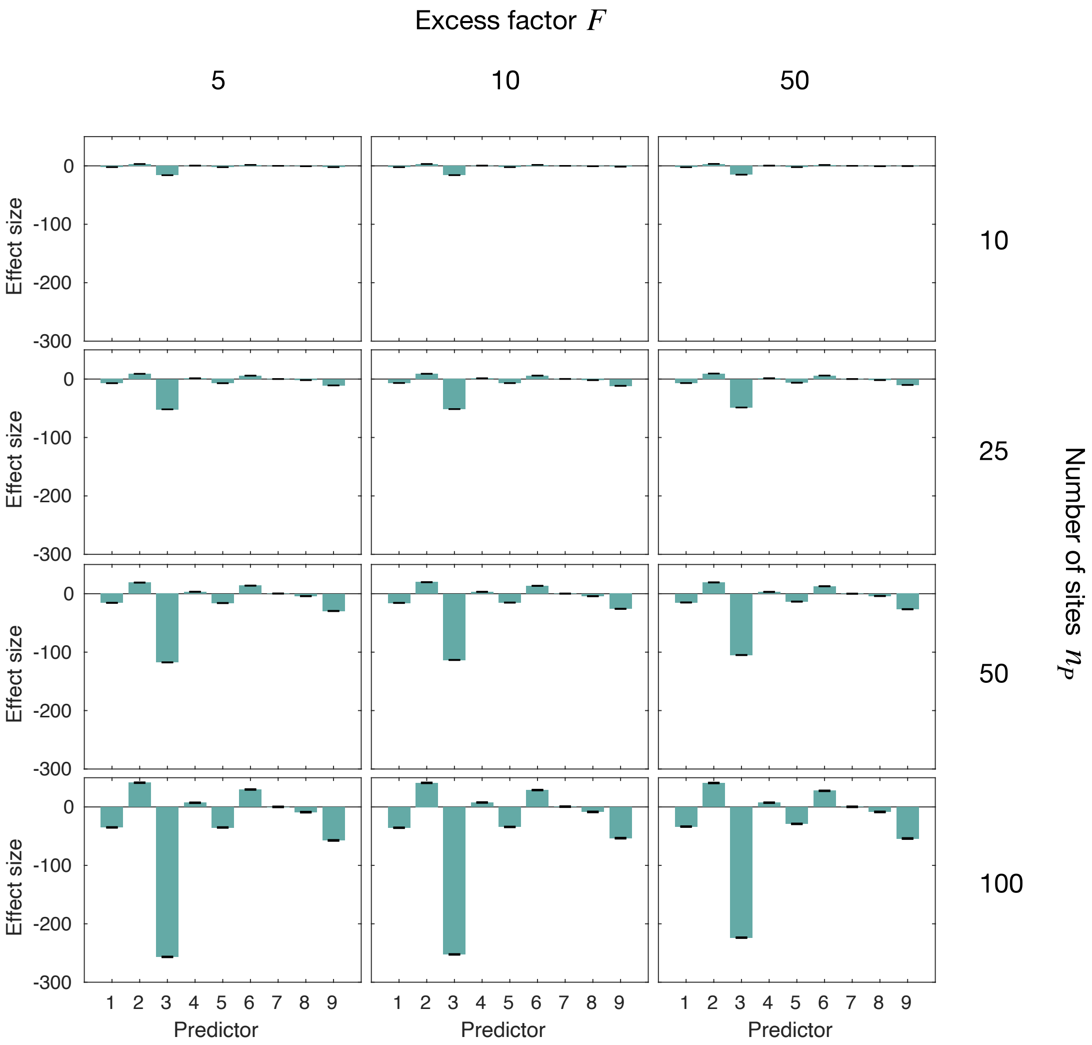

Fig. SI8. Effect sizes on A-sensitivities of regional biomass $\mathcal{S}[\mathcal{B}_\gamma]$ in a gradient of number of sites n_P and excess factor F. Predictors are foodweb temperature T (1), self limitation λ (2), dispersal ability $\hat{a} = \log_{10} a$ (3), $T \cdot \lambda$ (4), $T \times \hat{a}$ (5), $\lambda \times \hat{a}$ (6), T^2 (7), λ^2 (8), and $\hat{a}^2$ (9). Foodweb parameters are $n_S = 45$ and $C = 0.2$

2.3 Effects of local and regional stabilizing factors depending on foodweb topology

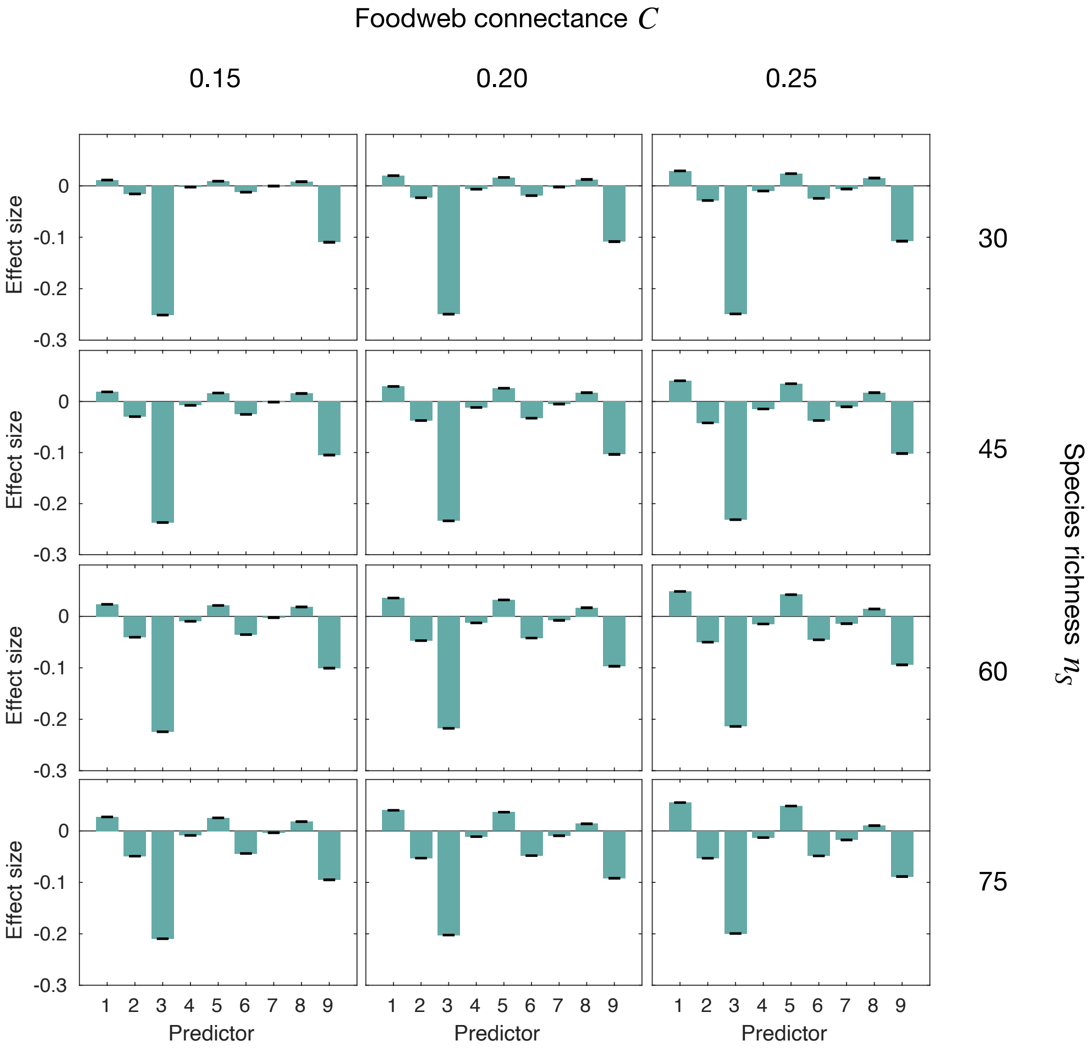

Fig. SI9. Effect sizes on A-sensitivities of local species persistence $\mathcal{S}[\mathcal{P}_\alpha]$ in a gradient of foodweb connectance C and species richness n_S in the regional pool. Predictors are foodweb temperature T (1), self limitation λ (2), dispersal ability $\hat{a} = \log_{10} a$ (3), $T \cdot \lambda$ (4), $T \times \hat{a}$ (5), $\lambda \times \hat{a}$ (6), T^2 (7), λ^2 (8), and $\hat{a}^2$ (9). Landscape parameters are $n_P = 50$ and $F = 50$

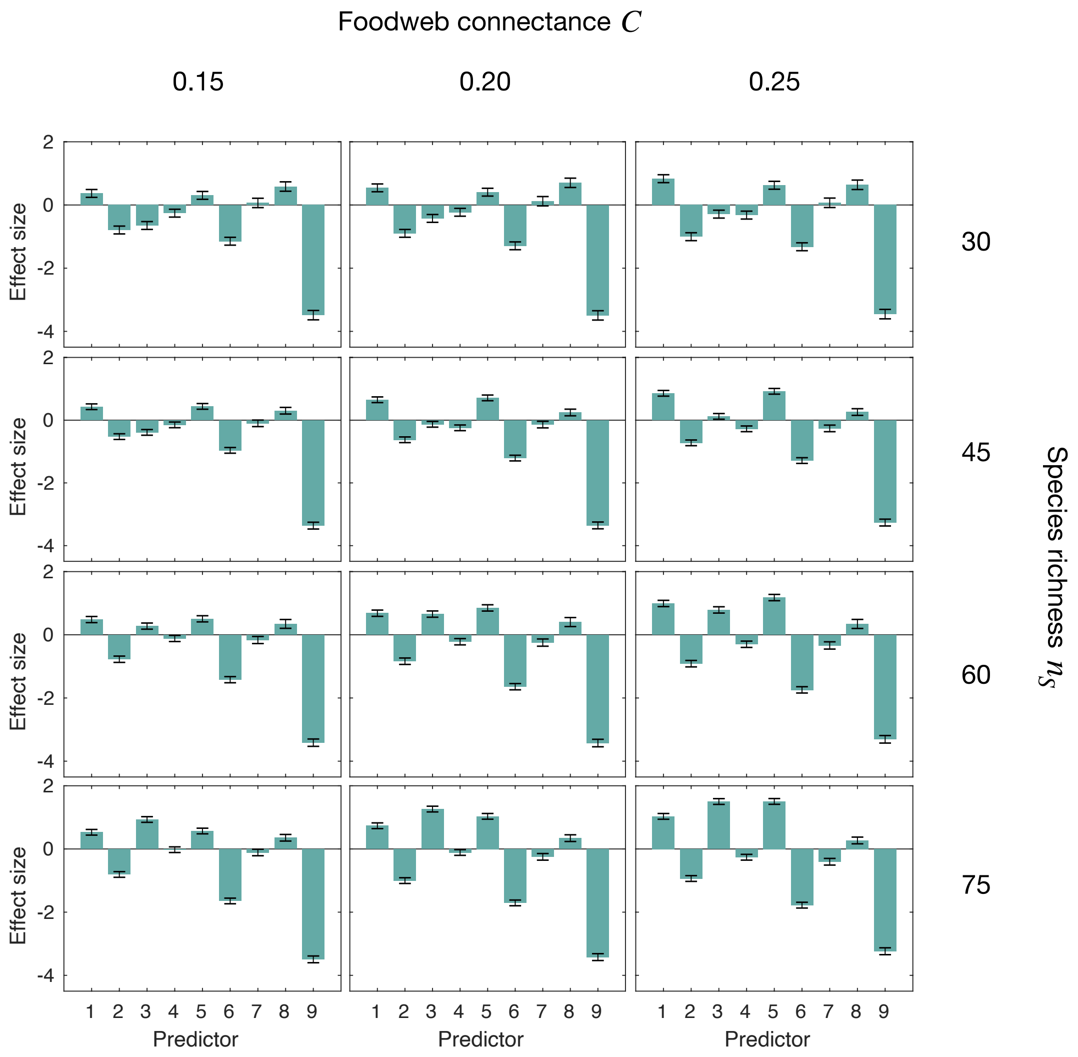

Fig. SI10. Effect sizes on A-sensitivities of among-site dissimilarity in species persistence $\mathcal{S}[\mathcal{P}_\beta]$ in a gradient of foodweb connectance C and species richness n_S in the regional pool. Predictors are foodweb temperature T (1), self limitation λ (2), dispersal ability $\hat{a} = \log_{10} a$ (3), $T \cdot \lambda$ (4), $T \times \hat{a}$ (5), $\lambda \times \hat{a}$ (6), T^2 (7), λ^2 (8), and $\hat{a}^2$ (9). Landscape parameters are $n_P = 50$ and $F = 50$

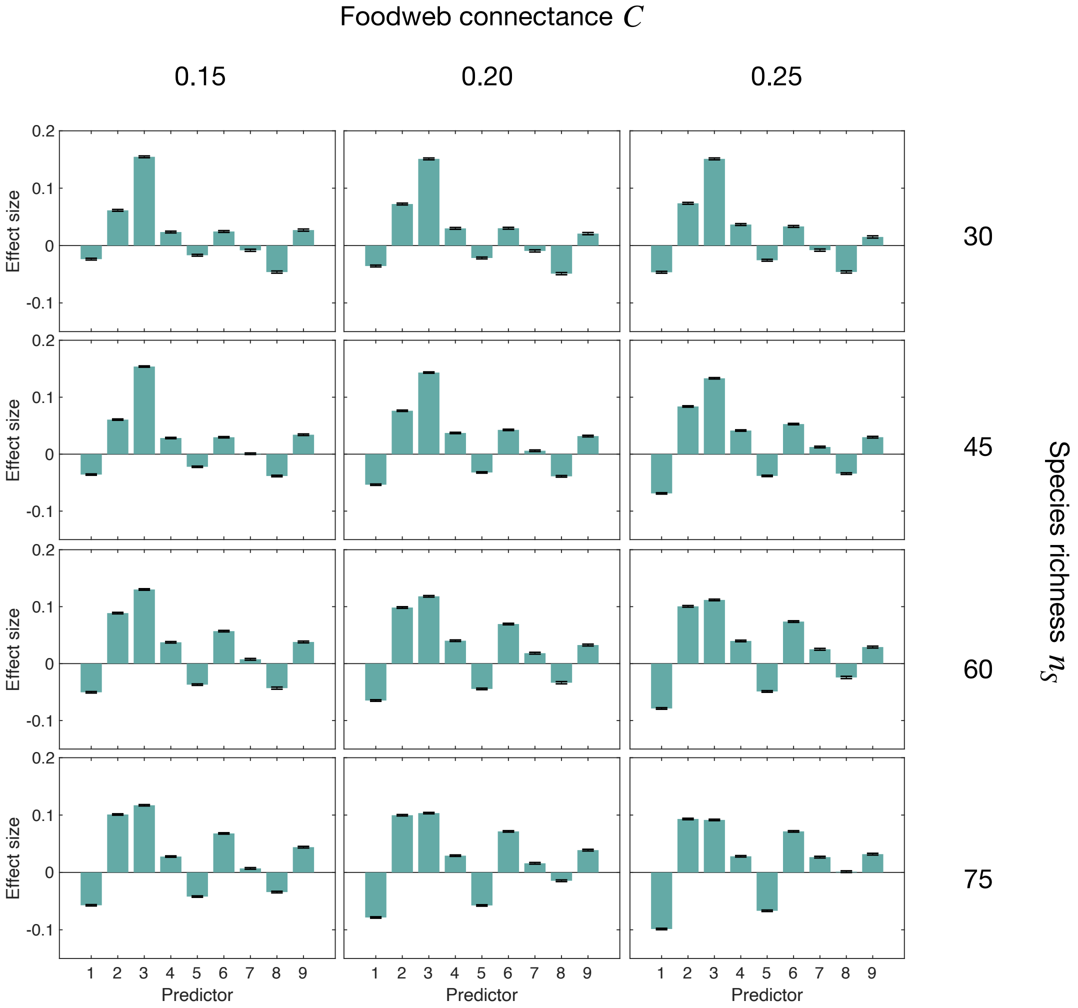

Fig. SI11. Effect sizes on A-sensitivities of regional species persistence $\mathcal{S}[\mathcal{P}_\gamma]$ in a gradient of foodweb connectance C and species richness n_S in the regional pool. Predictors are foodweb temperature T (1), self limitation λ (2), dispersal ability $\hat{a} = \log_{10} a$ (3), $T \cdot \lambda$ (4), $T \times \hat{a}$ (5), $\lambda \times \hat{a}$ (6), T^2 (7), λ^2 (8), and $\hat{a}^2$ (9). Landscape parameters are $n_P = 50$ and $F = 50$

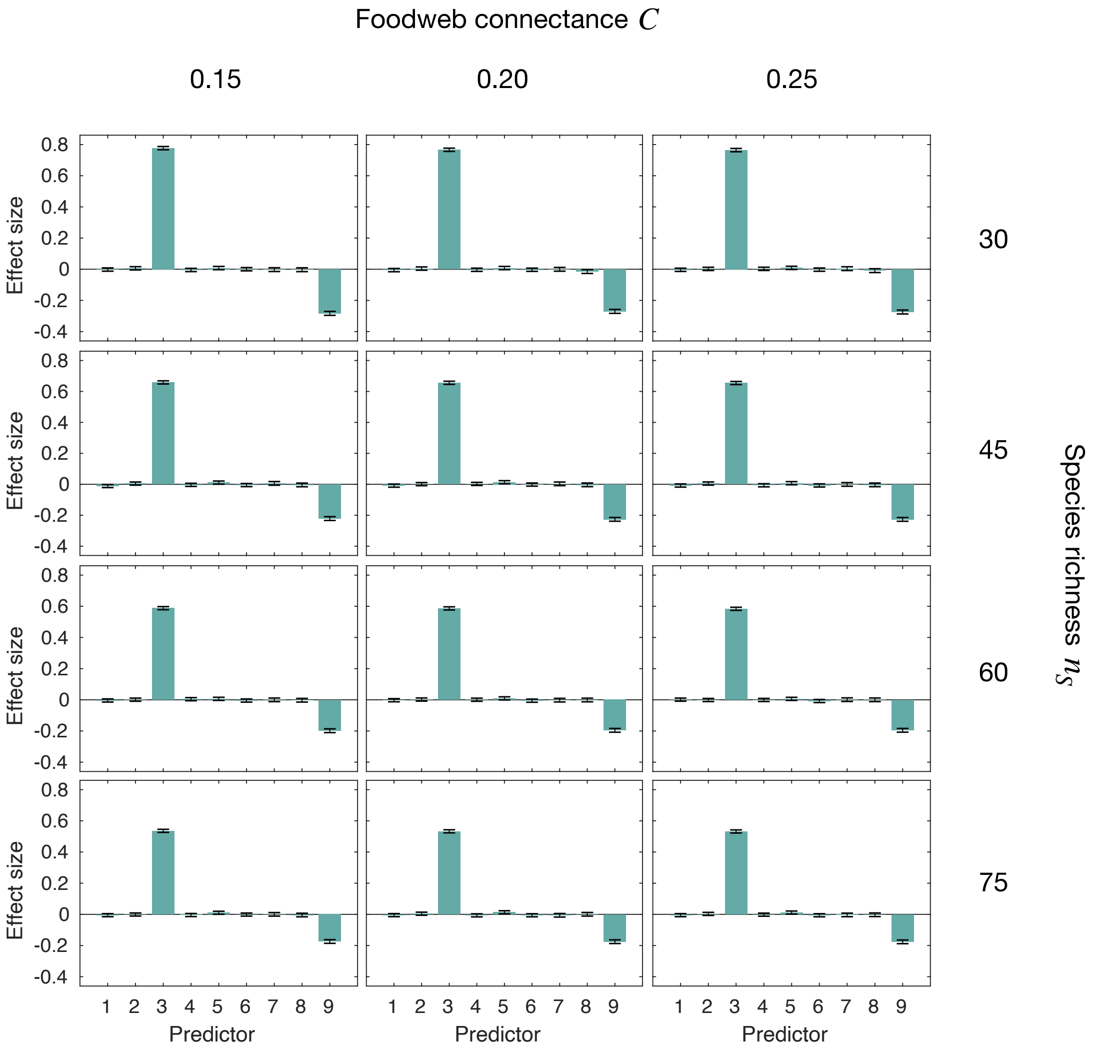

Fig. SI12. Effect sizes on A-sensitivities of among-site dissimilarity in community biomass $\mathcal{S}[\mathcal{B}_\beta]$ in a gradient of foodweb connectance C and species richness n_S in the regional pool. Predictors are foodweb temperature T (1), self limitation λ (2), dispersal ability $\hat{a} = \log_{10} a$ (3), $T \cdot \lambda$ (4), $T \times \hat{a}$ (5), $\lambda \times \hat{a}$ (6), T^2 (7), λ^2 (8), and $\hat{a}^2$ (9). Landscape parameters are $n_P = 50$ and $F = 50$

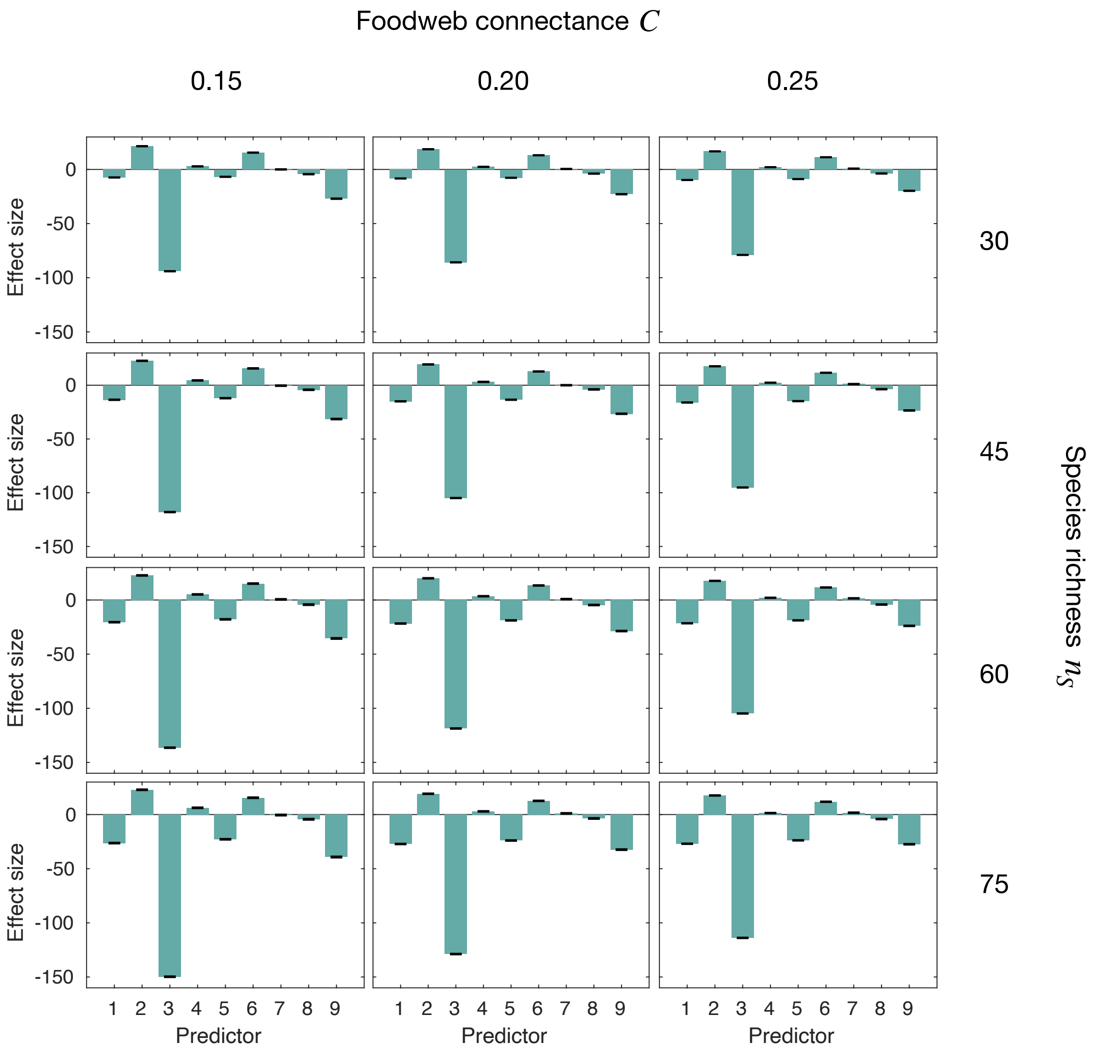

Fig. SI13. Effect sizes on A-sensitivities of regional biomass $\mathcal{S}[\mathcal{B}_\gamma]$ in a gradient of foodweb connectance C and species richness n_S in the regional pool. Predictors are foodweb temperature T (1), self limitation λ (2), dispersal ability $\hat{a} = \log_{10} a$ (3), $T \cdot \lambda$ (4), $T \times \hat{a}$ (5), $\lambda \times \hat{a}$ (6), T^2 (7), λ^2 (8), and $\hat{a}^2$ (9). Landscape parameters are $n_P = 50$ and $F = 50$

2.4 Sensitivities of $\mathcal{P}_\alpha$ and $\mathcal{P}_\gamma$

These figures depict metacommunity sensitivities as the changes in the distributions of $\mathcal{P}_\alpha$ and $\mathcal{P}_\gamma$ as sites' activation and deactivation asynchrony A increases from $A = 0$ to $A = 0.5$. For each parameter set, we run 50 replicates. However, for each data point, its coordinates represent the value of either $\mathcal{P}_\alpha$ or $\mathcal{P}_\gamma$ for the same foodweb and landscape but changing only A. The color of a dot represents the propensity to species persistence of a single local community (foodweb). The actual color depends on n_S, C, λ and T, and is looked up in Fig. SI2. Thus, yellow/blue dots represent high/low local persistence. Note that stable-prone local communities tend to produce metacommunities with high regional persistences. This is apparent by the clustering of yellow circles close to large $\mathcal{P}_\gamma$ values. By contrast, the blue circles, representing unstable-prone local communities are scattered over a larger $\mathcal{P}_\gamma$ range. All simulations were carried out for landscapes with $n_C = 5$ and $F = 50$, and for a timelapse of 5 years. The magnitudes of metacommunity sensitivities are the vertical distances between each dot and the identity line. Positive sensitivities, i.e., when the response variable increases with landscape variability, are reached when the data points lie above the identity line, and vice versa.

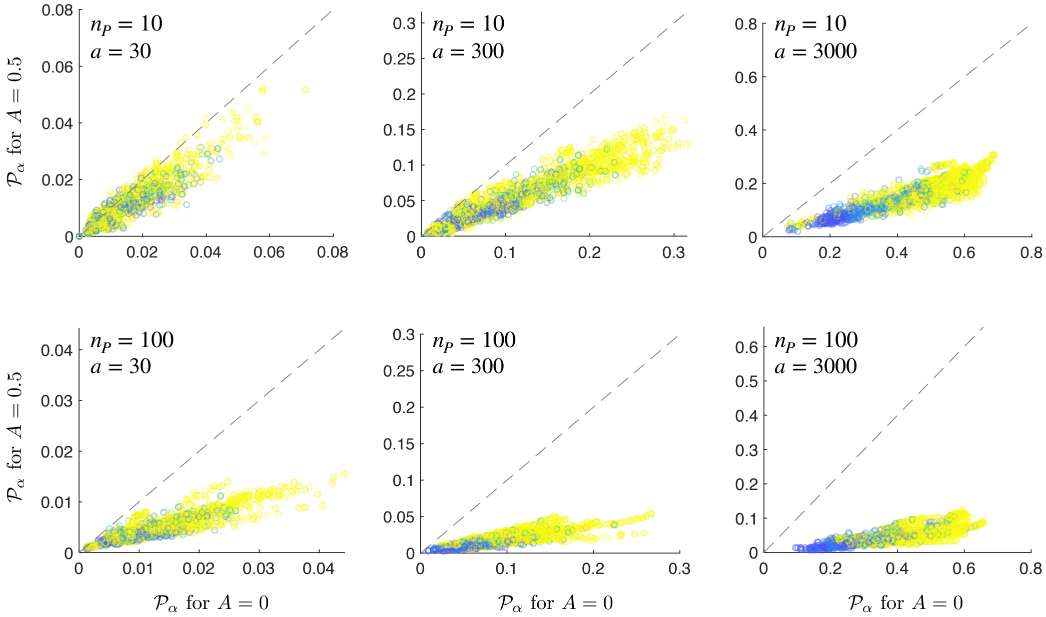

Fig. SI14. $\mathcal{P}_\alpha$ for $A = 0$ versus $A = 0.5$ and three levels of dispersal ability (a). Foodweb parameters were $n_S = 45$ and $C = 0.2$. The top and bottom rows correspond to scattered/spread and dense landscapes respectively.

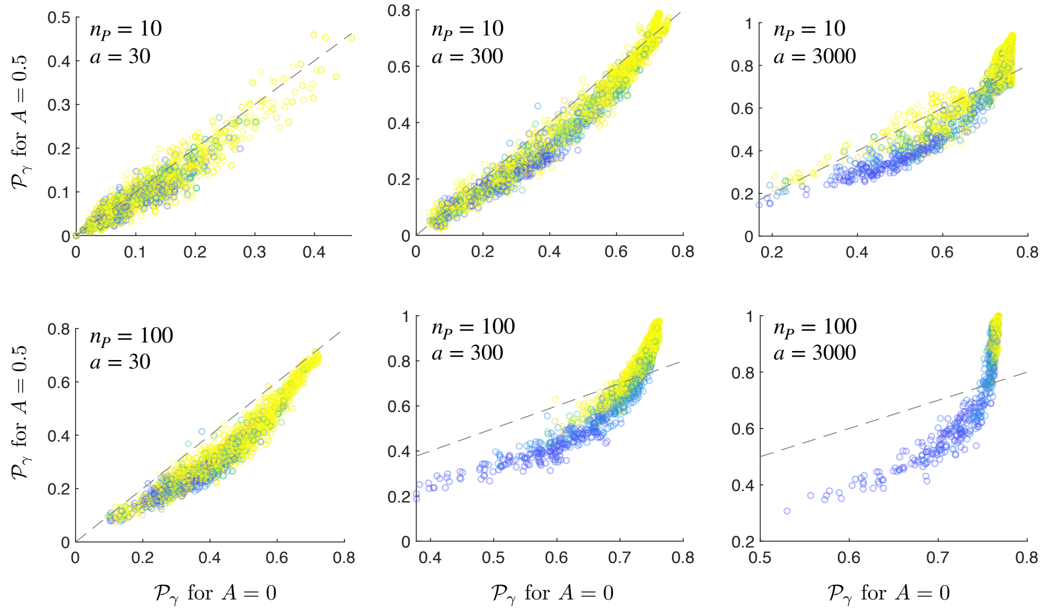

Fig. SI15. $\mathcal{P}_\gamma$ for $A = 0$ versus $A = 0.5$ and three levels of dispersal ability (a). Foodweb parameters were $n_S = 45$ and $C = 0.2$. The top and bottom rows correspond to scattered/spread and dense landscapes respectively.

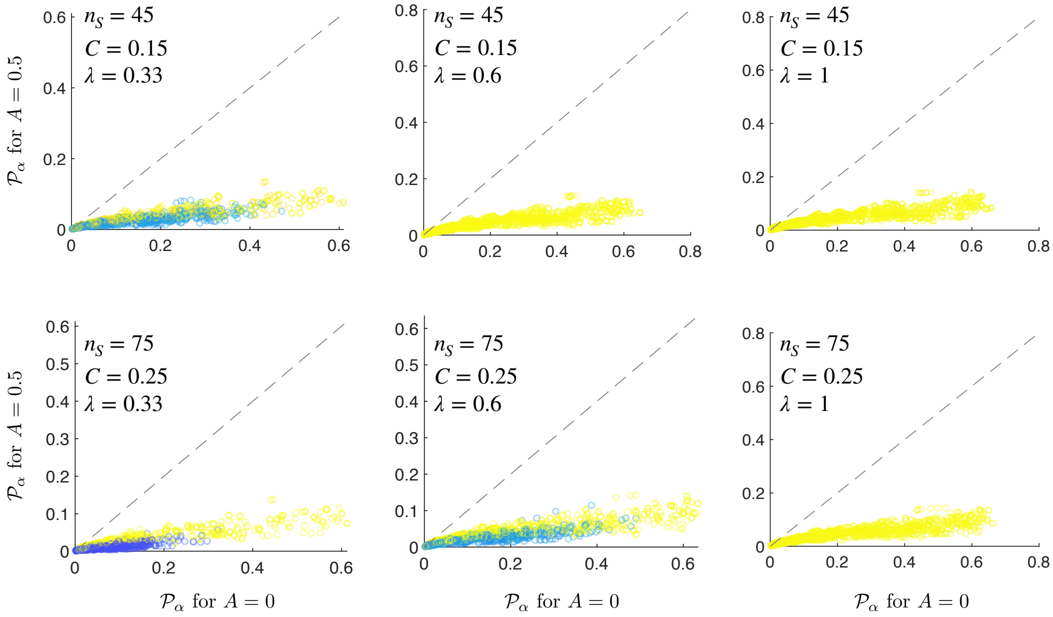

Fig. SI16. $\mathcal{P}_\alpha$ for $A = 0$ versus $A = 0.5$ with $n_P = 50$ and three levels of self limitation (λ). The top and bottom rows correspond to simpler and more complex foodwebs respectively.

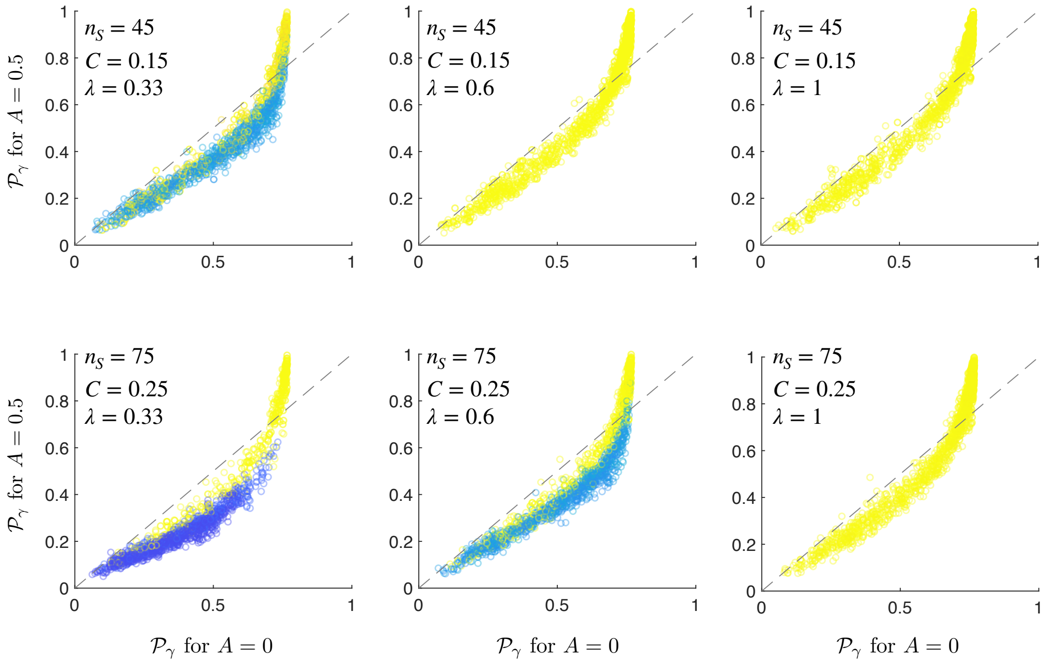

Fig. SI17. $\mathcal{P}_\gamma$ for $A = 0$ versus $A = 0.5$ with $n_P = 50$ and three levels of self limitation (λ). The top and bottom rows correspond to simpler and more complex foodwebs respectively.

2.5 Star experiment

This experiment simulated both stable-prone and unstable-prone local communities, on 10-point star landscapes. We used landscapes in which either all points or only three points can become active, mimicking low and high A values respectively. With higher A values, each temporal snapshot of the landscape graph turns sparser, thus implying fewer routes for dispersal among patches, which we approximate by allowing only three sites to be "active."

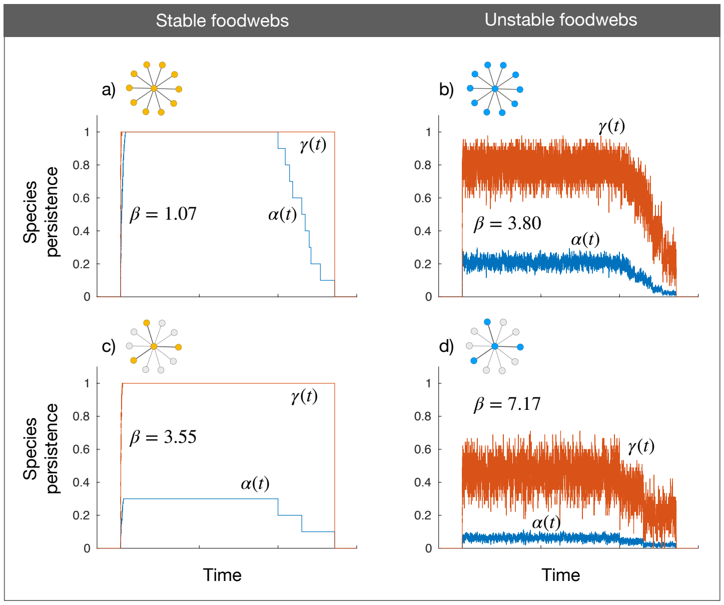

Fig. SI18. Time series of α (blue lines) and γ (red lines) diversity (species persistence) over a single simulated year, of multitrophic metacommunities on an idealized landscape of a 10-point star topology. The central site is the mainland. The time average of β diversity is shown within each plot. Parameters values are $a = 3000$, $n_S = 45$, $C = 0.2$. Plots a) and c) show the dynamics of stable communities ($T = 0$, $\lambda = 0.33$). Plots b) and d) show the dynamics of unstable communities ($T = 1$, $\lambda = 0.33$). In plots a) and b) all points (sites) are available for dispersal, representing highly synchronous landscapes. In plots c) and d) only 3 out of 10 points are available for dispersal, representing highly asynchronous landscapes.

For stable-prone local communities, all colonization attempts are successful and do not cause secondary extinctions. Thus, for sufficiently large values of a, we can assume that all active sites contain the full set of species. In this scenario, both $\mathcal{P}_\alpha$ and $\mathcal{P}_\gamma$ are maximal, while $\mathcal{P}_\beta$ is minimal. Increasing A (from Fig. SI18a to c) reduces $\mathcal{P}_\alpha$ simply by reducing the number of active sites. Conversely, if local communities are unstable-prone, two relevant competing processes take place. The first one includes successful colonization events that do not cause secondary extinctions. This process increases $\mathcal{P}_\alpha$ due to the direct introduction of a new species in the recipient community. In the second one, colonization attempts result in secondary extinctions, leading to reductions in $\mathcal{P}_\alpha$. The temporal alternation between these two processes causes the oscillations depicted in Figs. SI18b and d and prevents $\mathcal{P}_\alpha$ and $\mathcal{P}_\gamma$ from always reaching their maximum possible values. For $A = 0$, $\mathcal{P}_\alpha$ attains low values because local instability hampers

successful colonization events without secondary species extinctions. However, we do not observe a corresponding reduction in $\mathcal{P}_\gamma$, which shows there is a high among-site dissimilarity ($\mathcal{P}_\beta$). Increasing A (from Fig. SI18b to d) reduces $\mathcal{P}_\alpha$ because of reduction in active sites. However, the reduction in $\mathcal{P}_\gamma$ is relatively small because of the large values of $\mathcal{P}_\beta$. This small experiment captures the interplay between the LSFs and landscape asynchrony and how it shapes the biodiversity patterns observed in the full model.
